# Influencing tumor-associated macrophages in malignant melanoma with monoclonal antibodies

**DOI:** 10.1080/2162402X.2022.2127284

**Published:** 2022-10-03

**Authors:** Rebecca Adams, Gabriel Osborn, Bipashna Mukhia, Roman Laddach, Zena Willsmore, Alicia Chenoweth, Jenny L C Geh, Alastair D MacKenzie Ross, Ciaran Healy, Linda Barber, Sophia Tsoka, Victoria Sanz-Moreno, Katie E Lacy, Sophia N Karagiannis

**Affiliations:** a St. John’s Institute of Dermatology, School of Basic & Medical Biosciences, King’s College London, Guy’s Hospital, London, UK; b Department of Informatics, Faculty of Natural, Mathematical & Engineering Sciences, King’s College London, Bush House, London, UK; c Breast Cancer Now Research Unit, School of Cancer & Pharmaceutical Sciences, King’s College London, Innovation Hub, Guy’s Hospital, London, UK; d Department of Plastic Surgery at Guy’s, King’s, and St. Thomas’ Hospitals, London, UK; e School of Cancer & Pharmaceutical Sciences, King’s College London, Guy’s Hospital, London, UK; f Barts Cancer Institute, Queen Mary University of London, London, UK

**Keywords:** melanoma, macrophages, monoclonal antibodies, immunotherapy, checkpoint inhibitors, polarization, tumor microenvironment, Fc receptors

## Abstract

The application of monoclonal antibodies (mAbs) for the treatment of melanoma has significantly improved the clinical management of this malignancy over the last decade. Currently approved mAbs for melanoma enhance T cell effector immune responses by blocking immune checkpoint molecules PD-L1/PD-1 and CTLA-4. However, more than half of patients do not benefit from treatment. Targeting the prominent myeloid compartment within the tumor microenvironment, and in particular the ever-abundant tumor-associated macrophages (TAMs), may be a promising strategy to complement existing therapies and enhance treatment success. TAMs are a highly diverse and plastic subset of cells whose pro-tumor properties can support melanoma growth, angiogenesis and invasion. Understanding of their diversity, plasticity and multifaceted roles in cancer forms the basis for new promising TAM-centered treatment strategies. There are multiple mechanisms by which macrophages can be targeted with antibodies in a therapeutic setting, including by depletion, inhibition of specific pro-tumor properties, differential polarization to pro-inflammatory states and enhancement of antitumor immune functions. Here, we discuss TAMs in melanoma, their interactions with checkpoint inhibitor antibodies and emerging mAbs targeting different aspects of TAM biology and their potential to be translated to the clinic.

## Introduction: therapeutic challenges in melanoma and the emerging importance of tumor-infiltrating macrophages

Melanoma is the deadliest form of skin cancer with increasing incidence worldwide.^1,2^ Historically, surgery was the only definitive treatment. However, recent advances in systemic therapies include small-molecule drugs, BRAF and MEK inhibitors (BRAFi and MEKi), targeting the Mitogen-Activated Protein Kinase (MAPK) pathway, and immunotherapy in the form of immune checkpoint inhibitors. Checkpoint inhibitors block the regulatory functions of programmed death-1, PD-1 and its ligand PD-L1, and the cytotoxic T-lymphocyte-associated protein 4 (CTLA-4) on T cells. These have significantly improved patient outcomes.^3–5^ Despite success, current systemic therapies have multiple limitations. Patients may not respond to treatment at all, and, as of yet, there are no reliable biomarkers to identify such patients prior to treatment.^5^ Resistance to treatment often occurs, and presents a particular challenge with MAPK pathway inhibitors, whereby the majority of patients develop resistance within several months of treatment.^6^ Furthermore, some patients suffer severe adverse side effects to immunotherapy, meaning that they cannot continue with treatment.^5,7,8^ The success of checkpoint inhibition demonstrates that manipulation of the cancer immune environment can be achieved and that this can improve outcomes. Alongside, it is becoming increasingly clear that immune cell-targeted interventions need to be refined in order to create treatments that carry less risk to patients, and likely need to be complemented to enhance, prolong or maintain clinical efficacy.

As the archetypal immunogenic tumor, the correlation between immune cell infiltration and prognosis in melanoma depends upon the presence, as well as the nature, of the immune cells recruited in the tumor microenvironment (TME). A lymphocytic infiltrate is largely associated with a more favorable prognosis, while prognosis seems to worsen as the ratio of lymphocytes to myeloid cells, such as myeloid-derived suppressor cells (MDSCs), monocytes and macrophages, decreases.^9^ Tumor associated macrophages (TAMs) are found in many solid tumors, including melanoma, and their presence is associated with poorer clinical outcomes.^10–12^ It is increasingly appreciated that macrophages may harbor great potential as a future target of immunotherapy: they are abundant in tumor lesions; they contribute to many elements of the pathogenicity of melanomas; and they are highly adaptable, with attributes and functions that can be potentially manipulated in a therapeutic setting. There are multiple mechanisms by which these cells can be targeted for therapy. For example, a treatment could be developed to prevent their recruitment to, and their survival and growth within, the TME. Alternatively, therapeutic agents may be generated to alter macrophage pro-tumor functions, by mechanisms such as blocking immune inhibitory molecules, depleting regulatory macrophage subsets or repolarizing macrophages toward a more pro-inflammatory phenotype by engaging cell surface receptors.

Here we discuss the functions of TAMs, and we review monoclonal antibody approaches targeting different aspects of TAM biology in the context of melanoma. We focus on treatments currently in clinical trials and those which have the potential to be translated to clinical testing in the near future.

## Macrophages: an overview

Macrophages represent a diverse group of cells with multiple functions in health and disease.^13^ Historically, macrophages have been categorized into two broad subsets: M1 and M2.^14^ M1, or “classically activated” macrophages, are pro-inflammatory cells, polarized by lipopolysaccharide (LPS) and IFN-γ with important roles in mounting an innate response against microbial pathogens. Classical macrophages can phagocytose pathogens and foreign material and secrete inflammatory cytokines, such as IL-1β, IL-12, and TNF-α, IL-15, IL-6.^13,15^ Macrophages can also augment an adaptive immune response by presenting pathogenic antigens to the adaptive immune system.^16^ M2 “alternatively activated” macrophages exhibit a range of homeostatic and anti-inflammatory functions, involved in the resolution of inflammatory responses, promoting tissue repair and wound healing. They are polarized by Th2 cytokines, including IL-4 and IL-13, and express scavenger receptors, enabling the endocytosis of cellular and microbial debris.^14,17,18^ They secrete pro-angiogenic factors, such as VEGF and metalloproteinases, allowing remodeling of the extracellular matrix following an inflammatory reaction to restore homeostasis.^19,20^

Macrophages likely represent a spectrum of cell phenotypes with diverse functions, for example, more precise categorization has been proposed for M2 macrophages.^20,21^ This is based on how these cells can be polarized in vitro and the functions they demonstrate: M2a, or IL-4 macrophages, can be stimulated by IL-4, IL-13 and in the context of fungal and helminth infections; M2b, by immune complexes and LPS; M2c, by IL-10, and TGFβ and M2d, can be stimulated by IL-6 and adenosine. These different subgroups can be polarized *in vitro* but, *in vivo*, such distinct classification may not represent the true spectrum of these cells. TAMs are typically associated with M2-like phenotypic markers and functions, and yet high-dimensional flow cytometric analysis and immunohistochemistry evidence points to TAMs exhibiting markers and functions which overlap between both the pro- and anti-inflammatory subtypes.^22,23^ Aside from this, in some solid tumors, TAMs display antitumor functions without expressing canonical M1 markers.^24^ The data to-date thus suggest that TAMs cannot be clearly categorized by the existing subset classification: their phenotypes and functions are influenced by the environmental niche in which they reside, and they can have both pro- and antitumor attributes.

## Defining TAM subsets in Melanoma

Melanoma-associated macrophages can derive either from embryonic-derived tissue resident macrophages (Res-TAMs), recruited and maintained by colony-stimulating factor-1 (CSF-1) binding its receptor CSF1R,^25^ or through the recruitment of circulating monocytes via the CCL2/CCR2 chemokine pathway, which can differentiate into monocyte-derived macrophages (mo-TAMs).^26–28^ The exact contributions of each origin pool are still being explored, with a paucity of information on how macrophage ontogeny affects TAM function within melanoma. Much knowledge of ontogeny derives from mouse studies, due to the inability to undertake fate-mapping studies in humans. Alongside this, genetic similarities and a lack of markers that can help distinguish tissue-resident from monocyte-derived macrophages render further exploration into this area quite challenging.^29^ It appears that tissue resident macrophages are the first to be influenced by factors secreted from tumors. However, in the cancer types studied so far, the contribution of Res-TAMs and mo-TAMs appears organ specific:^29–31^ in pancreatic cancer models, Res-TAMs appeared to promote tumor growth; in human glioma samples mo-TAMs correlate with tumor grade; and in mouse models of lung cancer, macrophages of both origins appear to contribute to tumor growth. No such comparative studies of how macrophage origin can affect function have been carried out in melanoma.

Although ontogeny may contribute to the heterogeneity of TAMs, environmental factors also appear to have an important influence on their function, with the TME of melanoma able to polarize monocytes and macrophages to confer pro-tumor functions.^32,33^ In addition, tumor-associated polarization allows the exploitation of the anti-inflammatory, pro-repair functions of macrophages, which in turn can support melanoma growth, invasion and metastasis, promote melanoma cell viability and reduce immunogenic killing of cancer.^32^ This is manifested as the reported associations between M2-like phenotypic markers and poor prognosis.^11,12,23^

Within the TME, availability of oxygen and nutrients, as well as the gradient of secreted factors, varies and can influence the function of TAMs. Thus, TAMs within the TME demonstrate metabolic diversity, another factor that can impact on their function. For example, in areas of hypoxia, TAMs exhibit angiogenic and immunosuppressive functions, thought to be promoted by an upregulated expression REDD1, a negative regulator of mTOR.^34^ Alongside, single-cell RNA-sequence (sc-RNA-seq) analysis of human metastatic melanoma lesions suggested that TAM subsets with increased purine metabolism demonstrated reduced phagocytosis and antigen presentation abilities, as well as increased expression of angiogenic and immunosuppressive genes.^35^

Combining many of these models of macrophage classification, two large, high-dimensional sc-RNA-seq studies, across multiple cancer types ^36,37^ have recently been reviewed, with the authors using transcriptomic similarities to define TAM subsets that appear to be preserved across cancers.^38^ Subsets shared genetic features of macrophage origin, pro-tumor functions or metabolism. Although not all the seven defined subsets were found in melanoma samples, IFN-induced TAMs, regulatory TAMs, angiogenic TAMs, and a subset of TAMs which mimic resident tissue macrophages found in neighboring healthy tissue, were described. The gene signatures outlined may provide a basis for further research into the subsets found in melanoma and their contributions to disease and responses to therapy.

**Figure 1.**
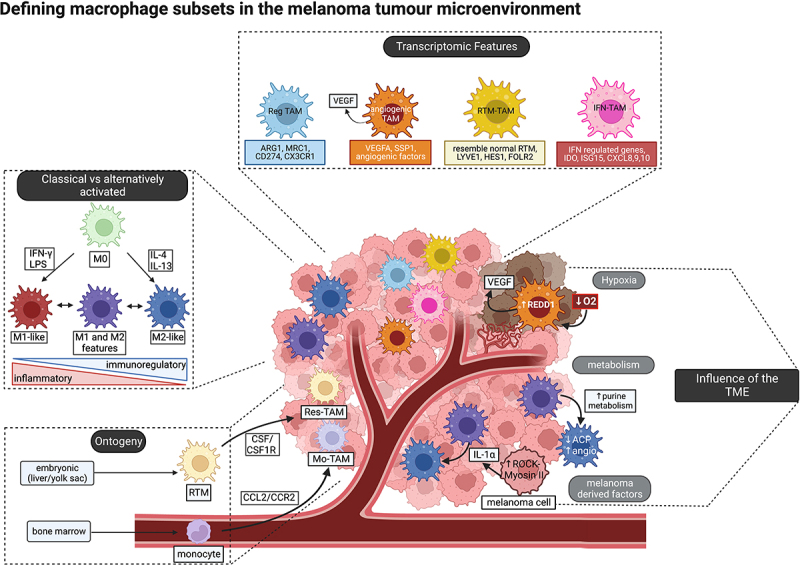
**Defining macrophage subsets in the melanoma tumor microenvironment** includes (1) using the classical/alternatively activated spectrum model (far left); (2) using differentially expressed genes to define subsets from single cell data (top); (3) understanding how the TME can promote subsets with specific functions (right); and (4) understanding how macrophage origin can determine phenotype and function (bottom left).

As demonstrated by the multiple theories on macrophage subsets reported over the last few decades (summarized in Figure 1), TAMs comprise highly plastic, heterogeneous and complex populations that warrant further exploration individually and as a whole.

## Macrophages and their contributions to melanoma

**Figure 2.**
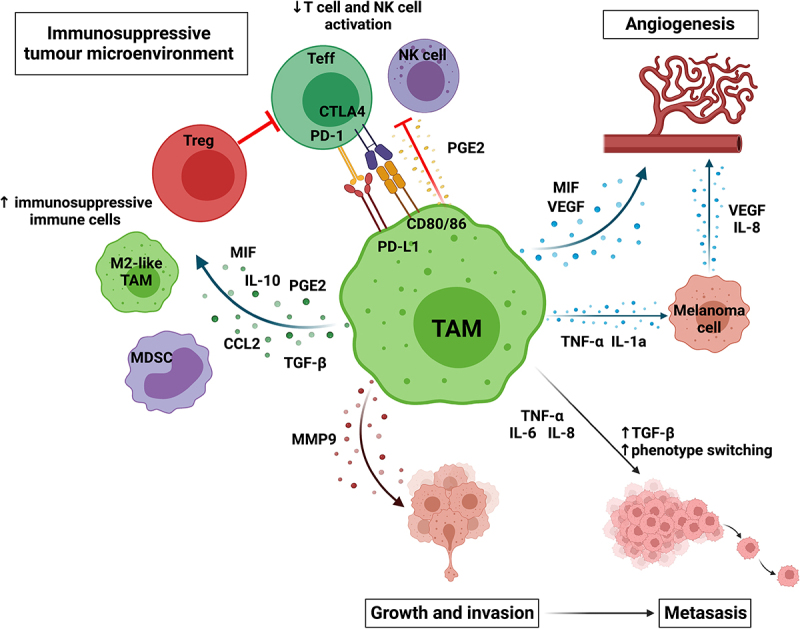
**TAMs can promote melanoma growth and progression** by creating an immunosuppressive immune environment by recruiting and maintaining immunosuppressive cells, such as Tregs, M2-like TAMs and MDSCs and reducing effector cells activation; promoting angiogenesis directly by secreting VEGF and MIF and indirectly through TNF-α and IL-1 α promoting angiogenesis by melanoma cells; enabling invasion by secreting metalloproteinases; and enabling metastasis by promoting the secretion of factors which increase phenotype switching (TGF-β), increase motility (IL-8), and increase invasion (IL-6). Created with BioRender.com.

Macrophages are thought to support melanoma progression through several mechanisms and positive feedback loops, illustrated in Figure 2. Firstly, TAMs can secrete factors, such as CCL2 and CCL1, which promote further macrophage recruitment and polarization, but also attract other immunosuppressive cells, such as MDSCs and tumor-promoting neutrophils^17,33,39,40^; and CCL17 and CCL22, which increase the recruitment of regulatory T cells (Tregs).^41^ Macrophages are known to further develop an immunosuppressive TME. They can reduce T cell effector function via the secretion of mediators such as prostaglandins,^42^ which, along with IL-10, can also promote the expansion of Treg populations and further recruitment and polarization of immunosuppressive TAMs.^43–45^ TAMs demonstrate reduced cytotoxic function, poor antigen-presenting ability,^42,46^ and express checkpoint molecule PD-L1, which has been reported to correlate with disease progression, modulated anti-cancer responses, and PD-1, which is associated with exhaustion, inability of TAMs to phagocytose tumor cells effectively, and impaired T cell activation.^47^

Contributing to this immunosuppressive environment, TAMs secrete migration inhibitory factor (MIF), which further supports polarization toward immunoregulatory TAMs and suppresses T cell activation.^48,49^ The importance of MIF has been demonstrated by MIF-CD74 signal blockade, which can drive CD8+ T cell infiltration and pro-inflammatory macrophage polarization in the TME, and can reduce hypoxia-inducible factor 1-alpha (HIF-1α) and PD-L1 expression by melanoma cells.^50,51^ Furthermore, macrophage-derived MIF is able to enhance melanoma growth and angiogenesis,^49^ along with other factors secreted by TAMs. An example is VEGF,^52^ which acts directly to promote new blood vessel growth, and secretion of TNF-α and IL-1α, which act indirectly to drive the secretion of pro-angiogenic factors by melanoma cells.^53^ Aside from angiogenesis, macrophages promote melanoma progression, as evidenced by their increased density at the invasive front of melanoma lesions,^54,55^ and promote metastasis. Invasiveness is increased by the secretion of metalloproteinases, such as MMP-9, which enable the remodeling of the extracellular matrix.^19^ Macrophages contribute to this by either secreting MMP-9 and expressing urokinase-type plasminogen activator (uPAR) directly or by promoting melanoma cells to secrete metalloproteinases and uPAR via the secretion of TNF-α.^56^ TAMs are thought to play an important role in melanoma metastasis, both in cancer phenotype switching, an epithelial to mesenchymal transition–like process, and the establishment of premetastatic niches, by promoting the survival and growth of cancer cells as they leave the primary tumor and invade distant tissue sites.^57–61^ Finally, the presence of TAMs correlates with resistance to both MAPKi and immune checkpoint inhibitors, with macrophages playing key roles in both the effector functions of such treatments and the mechanisms of resistance.^62^

## Monoclonal antibodies for the treatment of melanoma

The sea-change in melanoma therapy has arisen from MAPK pathway inhibitors, and monoclonal antibodies (mAbs) recognizing immune checkpoints. The use of mAbs in cancer therapy is an ever-growing field. mAbs consist of the Fragment antigen-binding (Fab) region, the portion of the antibody which binds target epitopes, and the Fragment crystallizable (Fc) region, the region that mediates interactions between the antibody and cells of the immune system.^63^ The Fc portion binds to Fc receptors (FcRs), expressed on a wide range of immune cells, including macrophages.^64^ Engagement of antibody Fc regions by macrophages is of significant importance in melanoma due to the abundance of macrophages in the TME.^65^ FcRs can either have activating downstream ITAM domain signaling, leading to pro-inflammatory effects, or, in the case of FcγRIIb, can be inhibitory via ITIM signaling, leading to regulation of cell activation and impaired effector functions.^63,66^ FcRs, their expression on immune cells and their downstream effects are summarized in Table 1. mAbs can be used to target cancer antigens, for example by the delivery of drug conjugates, as with Brentuximab in the treatment of lymphoma;^71^ using the Fab region to block important signaling pathways, for example, growth factors such as human epidermal growth factor receptor 2 (HER2), targeted by trastuzumab in breast cancer treatment,^72,73^ or by influencing immune cell function by engaging the Fc region on immune effector cells such as NK cells, monocytes and macrophages.^73^

**Table 1.** **Fc receptors expressed by human monocytic cells**, showing FcR structure, mouse orthologue, downstream signaling response, expression across different human immune cells and affinity to different immunoglobulin isotypes. Adapted from Bruhns et al.,^67,68^ Hogarth et al.,^69^ Bianchini et al.,^64^ and Chenoweth et al.^70^ Created with BioRender.com.

	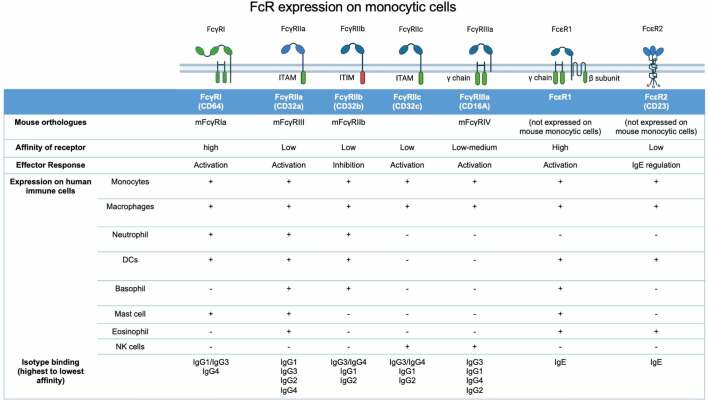

Within the context of melanoma, the current successful therapeutic use of mAbs take the form of checkpoint inhibitors, targeting CTLA-4, such as ipilimumab, or the PD-1/PD-L1 axis (e.g., anti-PD-1 antibodies pembrolizumab, nivolumab; or the anti-PD-L1 antibody atezolizumab). Checkpoint inhibitors enhance the immune response against melanoma by blocking molecular targets which usually limit or inhibit T cell responses.^74,75^ Although these therapies have demonstrated great efficacy in activating the T cell compartment within melanomas, they may also exert effects on the myeloid compartment of the TME.

## Interactions of TAMs with checkpoint inhibitors and targeted therapies: impact on treatment efficacy and resistance

PD-L1 is highly expressed by multiple cells including tumor and stromal cells, as well as TAMs within melanoma lesions,^76^ while the expression of PD-1 is widely described on the cell surface of activated T cells.^74^ Nivolumab (IgG4 mAb) or pembrolizumab (IgG4 mAb) binding to PD-1, or atezolizumab (IgG1 mAb) binding to PD-L1, inhibit PD-L1/PD-1 signaling, allowing T cell activation and reducing the number of exhausted T cells within the TME.^74,77^ Although immune checkpoint inhibitors such as anti-PD-1 mAbs promote antitumor activity, their efficacy may be limited by a feedback mechanism whereby T cells, reactivated by immunotherapy, secrete IFN-γ and TNF-α, which in turn can induce CSF-1 expression by melanoma cells.^78^ CSF-1 is an important factor that promotes the recruitment and growth of myeloid cells, including MDSCs and immunosuppressive TAMs.^25^ CSF-1 expression has been shown to correlate with TAM infiltration and poor prognosis and may contribute to anti-PD-1 therapy resistance.^78^

Further to this, macrophages have been shown to actively uptake anti-PD-1 mAbs from the surface of T cells. Using time-lapse intravital imaging in a mouse model of anti-PD-1 responsive cancer, anti-PD-1 mAb was rapidly removed from CD8+ T cells and transferred to TAMs in an Fc gamma receptor (FcγR) dependent mechanism.^79^ Anti-PD-1 mAbs used clinically to treat melanoma use the low-affinity IgG4 isotype instead of the higher affinity IgG1, in order to reduce antibody interaction with activatory FcRs expressed on myeloid cells (see Table 1). However, IgG4 retains high affinity to FcγRI. Cross-linking of PD-1 and FcγRI by anti-PD-1 mAbs can bring T cells and macrophages together, and lead to the killing of PD-1+ T cells by macrophages, as well as induce the secretion of anti-inflammatory cytokines, such as IL-10,^80^ thus reducing the therapeutic effect.

In contrast, an effector function mechanism may be involved in the antitumor functions of the anti-CTLA-4 antibody ipilimumab (IgG1 mAb). The checkpoint CTLA-4 is expressed by T cells, including high and constitutive expression by Tregs. While CD80 and CD86 binding to CD28 on T cells signals T cell activation, CTLA-4 competes with CD28 at the immune synapse, and inhibitory CTLA-4:CD80/CD86 interactions prevent T cell activation.^74^ Blocking this interaction with mAbs therefore results in T cell activation and enhanced effector function. Aside from this, ipilimumab-induced depletion of Tregs has been reported to occur when the Fab portion of the antibody binds CTLA-4, with simultaneous binding of the Fc portion to the FcγRs expressed on immune cells, including macrophages and monocytes. This is thought to induce antibody-dependent cellular cytotoxicity (ADCC) of Tregs.^81^ In patients who responded to ipilimumab, a study reported an increase in non-classical monocytes which express FcγRIIIA; higher ratio of FcγRIIIA+ CD68+ (M1-like) to CD163+ (M2-like) macrophages at baseline compared to non-responders; and decreased Treg infiltration after treatment. Corroborating this study, in mouse models of melanoma, macrophages in the TME which express mFcγRIV, an ortholog of human FcγRIIIA (see Table 1), are associated with antibody-mediated lysis of Treg cells.^82^ These studies demonstrate the interplay of different immune cells within the TME and the importance of taking into consideration the interactions between antibody Fc regions and immune cells such as macrophages when designing checkpoint inhibitor mAbs.

Aside from their interactions with mAbs in the context of checkpoint inhibition, TAMs have also been implicated in the development of BRAF inhibitor (BRAFi) resistance. Mutations in BRAF, which occur in 50% of melanoma, lead to the molecule being constitutively activated enabling multiple downstream pro-tumor functions. BRAFi blocks this activated pathway but can paradoxically activate the MAPK pathway in cells that lack the BRAF mutation, including TAMs. This paradoxical activation can result in the promotion of pro-tumor TAM subsets which contribute further to BRAFi resistance through the secretion of macrophage-derived growth factors, such as VEGF, which in turn can support the reactivation of the MAPK pathway in tumor cells, thus enhancing melanoma growth.^52,83^

## Therapeutic strategies targeting TAMs in melanoma

**Figure 3.**
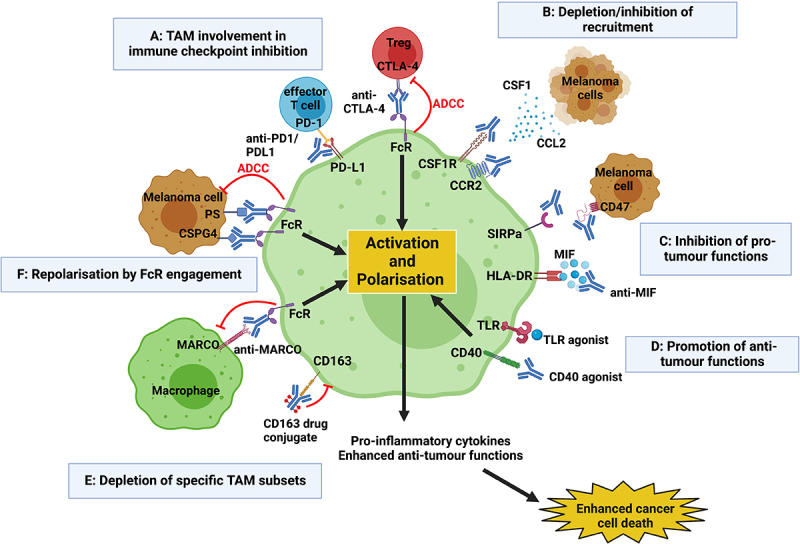
**Current techniques to therapeutically target TAMs** include (a) involvement of TAMs in current immune checkpoint inhibition; (b) reducing recruitment of macrophages to the TME, e.g. by blocking CCR2/CCL2 and CSF-1/CSFR1 pathways; (c) inhibiting pro-tumor functions of TAMS; (d) promoting antitumor functions of TAMS; (e) depleting specific pro-tumor subsets of TAMs; (f) repolarizing TAMs through the engagement of their FcRs, triggering downstream activation, increased secretion of pro-inflammatory cytokines and enhanced cancer cell death. Created with BioRender.com.

The complex interaction between different cell types in the TME, including cancer, stromal and immune cells, can drive tumor initiation and progression. This includes the interactions not only between tumor and immune cells but also between different immune compartments. Therefore, a multifaceted therapeutic approach, where key interactions that influence outcomes are disrupted, may translate into more effective treatments. It is clear from the studies above that simultaneous targeting of both the lymphoid compartment within melanoma, i.e., by checkpoint inhibition, and the myeloid compartment of the TME, may enhance the effectiveness of both treatments, increasing T cell effector function and ultimately leading to more cancer cell death. Different treatment approaches are being designed to target macrophages and their functions, with therapies aiming to deplete, limit the recruitment, influence the function of or re-educate macrophages or subsets of these cells, and are summarized in Figure 3. After several promising preclinical studies, some therapies have now progressed to clinical trials, as outlined in Table 2, and these approaches are discussed in the following sections.

**Table 2.** Examples of current and ongoing clinical trials targeting macrophages in melanoma.

Drug Name	Type	Target	Cancer Type	Phase	Status	Study Design	Route of Drug Administration	Reference
Imalumab	mAb	MIF	Solid tumors	1	Complete	Anti-MIF antibody monotherapy	Intravenous	NCT01765790
PLX3397	KIT inhibitor	CSF1R	Melanoma	1/2	Active	PLX3397 monotherapy	Oral	NCT02975700
PLX3397	KIT inhibitor	CSF1R	Mucosal and acral melanoma	2	Complete	PLX3397 monotherapy	Oral	NCT02071940
LY3022855	mAb	CSF1R	Melanoma	1/2	Active	LY3022855 + BRAFi + MEKi	Intravenous	NCT03101254
APX005M Cabiralizumab	mAb (IgG1) mAb (IgG4)	CD40 CSF1R	Melanoma	1/1b	Active	AP005M + Cabiralizumab + nivolumab	Intravenous Intravenous	NCT03502330
APX005M	mAb (IgG1)	CD40	Melanoma	1/2	Active	APX005M + Pembrolizumab	Intratumoral	NCT02706353
APX005M	mAb (IgG1)	CD40	Melanoma	2	Active	APX005M + /- radiotherapy	Intravenous	NCT04337931
SEA-CD40	mAb (IgG1)	CD40	Melanoma	2	Active	SEA-CD40 + pembrolizumab	Intravenous	NCT04993677
CDX-1140 Poly-ICLC	mAb (IgG2) TLR3 agonist	CD40 TLR3	Melanoma	1/2	Active	Melanoma mutated neoantigen peptide vaccine + CD40 agonist + TLR3 agonist	Intratumoral subcutaneous/intradermal	NCT04364230
PolyICLC Resiquimod	TLR3 agonist TLR7 agonist	TLR3 TLR7	Melanoma	1/2	Active	Peptide vaccine + tetanus vaccine ± PolyICLC ± resiquimod +/1 IFA	Intratumoral subcutaneous/intradermal	NCT02126579
MGN1703	TLR9 agonist	TLR9	Melanoma	1	Active	MGN1703 + Ipilimumab	Subcutaneous and intratumoural	NCT02668770
CMP-001	TLR9 agonist	TLR9	Melanoma	2	Active	CMP-001 + Nivolumab	Intratumoural subcutaneous/intradermal	NCT04698187
CMP-001	TLR9 agonist	TL9	Melanoma	2	Active	CMP-001 + Nivolumab	Intratumoural subcutaneous/intradermal	NCT03618641
CMP-001	TLR9 agonist	TLR9	Melanoma	2/3	Active	CMP-001 + Nivolumab vs nivolumab monotherapy	Intratumoral	NCT04695977

Data extracted from ClinicalTrials.gov.

Abbreviations: mAb, monoclonal antibody; KIT, receptor tyrosine kinase; TLR, toll like receptor; MIF, migration inhibitory factor; CSF1R, colony-stimulating factor 1 receptor; BRAFi, BRAF inhibitor; MEKi, MEK inhibitor.

### Targeting macrophage recruitment to the TME

Chemoattractant signals in the TME may facilitate higher rates of macrophage infiltration. Hence, targeting molecules involved in monocyte/macrophage recruitment has been investigated for tumor types where higher macrophage infiltration has been correlated with enhanced tumor progression and/or poorer prognosis.^11,12^ As discussed above, CSF-1 signaling through CSF1R is essential for the recruitment of myeloid cells to the TME, myeloid cell growth and it has been shown to contribute to maintaining tissue resident macrophage populations.^25^ In several *in vivo* models of melanoma, the tyrosine kinase inhibitor of CSF1R, PLX3397, has been shown to reduce macrophage recruitment to tumors as well as to shift resident macrophages toward a pro-inflammatory phenotype. This coincides with improved T cell infiltration, enhanced cytotoxic functions of infiltrating T cells, and tumor shrinkage. PLX3397 has also been shown to augment the effects of BRAFi ^84^ and of adoptive cell therapy.^85^ This agent is currently in phase 1/2 trials as a monotherapy in melanoma (NCT02975700, NCT02071940, see Table 2). Despite this apparent progress, it may be worth noting that two clinical trials of PLX3397 in combination with the BRAFi vemurafenib (NCT01826448) and the anti-PD-1 mAb pembrolizumab (NCT02452424) to treat melanoma were terminated due to lack of clinical efficacy.

Building on this approach, mAbs have been used to block CSF1R. Emactuzumab, a fully humanized IgG1 mAb against CSF1R, is currently undergoing clinical trials for solid tumors, including melanoma refractory to other treatments.^86^ The early data from the emactuzumab trial suggest that although blocking CSF1R on its own was able to reduce infiltration of immunosuppressive macrophages into the TME, this did not correlate with objective clinical responses.^87^ Another concern is that blocking CSF1R would not be selective for TAMs and it may be problematic to deplete all myeloid cells for a prolonged period of time, since subsets of these may be required for effective antitumor responses.^88^ Aside from emactuzumab, LY3022855, a recombinant human IgG1 mAb targeting CSF1R, is in phase 1 trials in combination with MAPK pathway inhibitors (NCT03101254, see Table 2). Macrophages are key players in the development of BRAFi resistance and such a combination may offer the chance to improve the longevity of an effective BRAFi response.

Due to the importance of the CCR2/CCL2 chemokine signaling in recruiting immunosuppressive monocytes and TAMs, there are ongoing trials to establish the anti-cancer effects of monoclonal antibodies designed against this pathway.^15,88^ When initially trialed, the fully human IgG1 anti-CCL2 antibody carlumab failed to demonstrate anticancer activity when used as a stand-alone treatment for solid tumors refractory to treatment,^89^ or when combined with chemotherapy.^90^ This exemplifies the difficulties in exploiting chemokine pathways therapeutically: there is much redundancy, lack of sufficient specificity for the tumor microenvironment and overlap within different chemokine pathways. These trials predate immune checkpoint inhibition, and it is possible that a combination of immune targeting approaches is required to promote a clinically significant anticancer response. Since the advent of checkpoint inhibition, preclinical studies demonstrated that combining checkpoint blockade with CCL2/CCR2 inhibition improved responses over checkpoint blockade alone treatment in murine models of melanoma.^91^ The improved efficacy of this approach may be due to simultaneous targeting of both the lymphoid and myeloid compartments of the TME. However, when plozalizumab, a humanized IgG1 mAb directed against CCR2, was combined with nivolumab, the trial was stopped early due to lack of discernible benefit (NCT02723006).

### Fine-tuning the targeting of tumor-associated macrophages

The above strategies have failed to make progress into later stage clinical trials (see Supplementary Table 1), and this may be due to their blunt nature: they deplete or inhibit the recruitment of all TAMs. As mentioned, macrophages are a highly plastic and diverse population of cells and although the pro-tumor functions of TAMs have been well characterized, these cells can also engender antitumor properties. These attributes are largely associated with the pro-inflammatory macrophage phenotype and include the secretion of pro-inflammatory cytokines, antigen presentation to T cells and direct killing of cancer cells.^17^ Alongside, macrophages have important effector roles in currently approved immunotherapies, as discussed above.^81^ It is therefore worth considering an approach whereby specific subsets of TAMs, especially those with immunosuppressive functions may be targeted. The drawback of this may be that often the same TAM subsets are able to confer both pro- and antitumor functions concurrently.

There are several strategies being explored to target macrophages more specifically, and this includes targeting pro-tumoral molecules secreted by TAMs, agonists to cell surface receptors that can either promote anticancer effector function or inhibit immunosuppressive functions and finally, by aiming to target molecules whose expression is likely restricted to the most immune suppressive TAMs.

### Inhibiting pro-tumor functions of macrophages: MIF

MIF is thought to play an important role in the polarization of macrophages toward more immunoregulatory, pro-tumor subsets.^49,50^ Multiple mechanisms of inhibiting MIF have been evaluated in preclinical studies, including the inhibitor 4-IPP, a small peptide which acts as a CD74 antagonist, thus preventing MIF-CD74 signaling. It has been reported that blocking MIF can alter the function of macrophages, and thus the nature of the melanoma TME.^49^ In mouse models of melanoma and melanoma lung metastasis, inhibiting MIF signaling resulted in reduced tumor burden and in increased numbers of pro-inflammatory TAMs with reduced pro-angiogenic capacity.^50^ When trialed in combination with anti-CTLA-4 and anti-PD-L1 antibodies in checkpoint inhibitor resistant in vivo models, a small-molecule inhibitor of MIF led to decreased tumor burden and prolonged survival, with greater infiltration of both pro-inflammatory TAMs and cytotoxic lymphocytes.^51^ This demonstrates a promising proof of concept: by making the TME more pro-inflammatory, the effectiveness of current therapies can be augmented. Although neither of these mechanisms of inhibiting MIF have yet been translated to clinical trials in melanoma, a phase 1 clinical study of an anti-MIF mAb in solid tumors has recently been completed, with results awaited (NCT01765790, Table 2).

### Promoting antitumor macrophage functions

#### The SIRPα/CD47 axis

When bound to signal-regulatory protein alpha (SIRPα) on the surface of myeloid cells, CD47 expressed in normal tissues acts as tolerogenic marker, inhibiting phagocytosis and preventing destruction of “self” cells. CD47 is essential for the maintenance of hematopoietic stem cells, erythrocytes and platelets, but is also upregulated by cancer cells as a means of immune escape.^92^ Targeting CD47 and blocking its signaling through SIPRα, for example with a mAb, could overcome this tolerogenic pathway, increase the ability of phagocytes, such as macrophages, to phagocytose cancer cells and facilitate or enhance cancer cell killing.^92,93^ This is of great interest in melanoma, due to the abundance of macrophages within the TME. However, there are concerns with toxicity: the expression of CD47 is ubiquitous, but it is especially expressed by vascular endothelial cells. In earlier clinical trials, targeting CD47 resulted in common side effects of anemia, thrombocytopenia, lymphopenia and hyperbilirubinemia.^93^ One anti-CD47 mAb, magrolimab (humanized IgG4 mAb), has made it through to phase 3 trials in patients with myelodysplastic syndrome (ENHANCE/NCT04313881), but anti-CD47 mAbs have not yet had such success in melanoma. Two phase 1 trials were initiated to study an IgG4 mAb against CD47 in melanoma, which was conjugated to a (not yet fully elucidated) toxin, as a monotherapy and in combination with other immunotherapies including anti-PD-1 (NCT03957096, NCT02890368). Unfortunately, both these trials were terminated, with little information for the reasons for termination and no data have been published. Ongoing trials include early phase 1/2 trials exploring CD47 as a target for antibody–drug conjugates, but also some studies aimed at blocking the CD47-SIRPα pathway in unresectable solid malignancies. There are no results from these trials published to date.

To overcome the toxicities associated with targeting the ubiquitous CD47, targeting SIRPα, may be a viable alternative as expression is limited to myeloid cells. SIRPα-targeting antibodies, hAB21, a humanized IgG1 mAb, with an inactive Fc domain, and 1H9, a humanized IgG1 mAb, have shown, in a variety of mouse models, to be an effective adjuvant to immunotherapies against highly immunogenic tumors, including melanoma.^94,95^ Although not yet in clinical trials for melanoma, two anti-SIRPα mAbs are currently in phase 1/2 clinical trials for solid tumors: BI 754091, a humanized IgG4 is being trialed in colorectal and endometrial cancer (NCT03990233) and BI 765063, a humanized IgG4, is being tested in head and neck and liver cancer (NCT05249426).

#### CD40/CD40L

CD40 is a target of significant interest in many solid cancers, including melanoma. CD40 is expressed on antigen presenting cells, including macrophages. Once bound by CD40 ligand on T cells, it can stimulate macrophages to produce pro-inflammatory cytokines, as well as to engage in direct target cell killing.^96^ Several clinical trials have looked at the potential of CD40 agonists as a monotherapy for treatment-resistant solid tumors. For example, a trial of selicrelumab, a human IgG2 agonistic mAb against CD40, in 15 patients with melanoma, demonstrated a partial response in 4 patients, some of which were long-lasting responses.^97^ More recently, selicrelumab in combination with CTLA-4 inhibition showed an overall response rate of 27.3% compared with 10.7% in the anti-CTLA-4 only arm.^98–100^ There are currently no ongoing trials further exploring selicrelumab in melanoma. APX005M and SEA-CD40 are both humanized IgG1 agonistic CD40 mAbs currently tested in melanoma in multiple phase 2 trials, in combination with pembrolizumab (NCT02706353, NCT04337931, NCT04993677, see Table 2). Furthermore, CDX-1140 is a human IgG2 mAb being trialed in combination with a melanoma peptide vaccine and a TLR agonist (NCT04364230). Results from these trials are awaited.

More recently, the focus on CD40 agonists has been directed to combinations with myeloid cell depletion strategies, such as anti-CSF1R. Preclinical studies demonstrated that when CD40 agonists were combined with CSF1R inhibitors in murine models of melanoma, TAMs secreted pro-inflammatory cytokines, such as IL-1b and IL-27, and reduced their expression of MMP9, suggesting an effective, albeit temporary, reprogramming.^96,101^ In addition to this, an enhanced T cell response against melanoma was reported, which was macrophage dependent.^96,101^ These data suggest that the reprogramming of TAMs has the potential to play an important role in promoting an anticancer TME, which in turn enables improved T cell effector function. The combination may seem counterintuitive, in that by using anti-CSF1R and CD40 agonism, TAMs are being both depleted and reprogrammed. However, it has been suggested that activation occurs rapidly with CD40 agonism while depletion of TAMs is much slower, and these mechanisms may occur alongside a preferential recruitment of pro-inflammatory TAMs in tumors exposed to this combination of therapies.^101^ This concept is being taken forward with an ongoing phase 1 clinical trial exploring the combination of the APX005M CD40 agonistic mAb with the anti-CSF1R IgG4 mAb cabiralizumab, with and without nivolumab (NCT03502330, see Table 2).

#### TLRs

Toll-like-receptors (TLRs), expressed on immune cells including macrophages, recognize pathogen-associated and damage-associated molecular patterns, and these interactions activate pro-inflammatory immune responses to ultimately result in the immune clearance of pathogens.^102^ TLR agonists have a potent pro-inflammatory effect and are currently used as first line therapy in superficial non-melanoma skin cancers and can be used as treatment for lentigo maligna, or melanoma in situ, in cosmetically sensitive areas. Applied topically, their side effects are limited; however, systemic application is associated with severe toxicity, including cytokine storm, as well as with the development of tolerance over time.^103^ Preclinical data suggest that TLR agonists can increase the presence of pro-inflammatory TAMs in melanoma lesions, which in turn modifies the TME to exert anticancer effects.^104,105^ TLR agonists can also enhance concurrent therapies, including anti-cancer antigen mAbs,^104^ checkpoint inhibitors ^106^ and BRAF inhibitors.^107^ Designing TLR agonists that are retained in the TME after intratumoral injection may be one way of enhancing their efficacy while limiting toxicities of such drugs. The lipophilic TLR7/8 agonist MEDI9197, has been designed to be retained at the site of injection and appears to not only polarize macrophages in in vitro studies but when tested in mouse models, recruits and activates immune cells, including CD8+ T cells and NK cells, while creating a more inflammatory TME.^108^

This ability to turn a “cold tumor” into a “hot tumor”, with a dense immune infiltrate, makes TLR agonists an attractive adjunct to other immune therapies. In one study using a checkpoint inhibitor-resistant mouse model of melanoma, TLR agonists in combination with CD40L, injected intratumorally, demonstrated an abscopal affect, whereby administration of CD40L and TLR agonist potentiated PD-1 blockade, not only locally but also at distant sites.^106^ Combination therapy of a CD40 agonist and a TLR3 agonist has reached clinical trials, and the TLR9 agonist CMP-001 has reached phase 2/3 trials (see Table 2).

### Depleting specific macrophage subsets

#### CD163

The expression of CD163 is associated with immunoregulatory TAMS with pro-tumoral functions. The presence of CD163+ macrophages in melanoma lesions is associated with poorer prognosis.^23^ CD163 is upregulated by cytokines such as IL-6 and IL-10 found in the melanoma TME, and downregulated by pro-inflammatory cytokines, and M1-like polarization signals, such as IFN-γ and TNF-α.^109^ Targeting CD163 may therefore represent a strategy to eliminate specific macrophage subsets which are known to contribute to melanoma progression. Etzerodt *et al*. developed an anti-CD163 IgG mAb conjugated with lipid nanoparticles loaded with the cytotoxic agent doxorubicin.^109^ In a mouse model of BRAF-mutant melanoma, the group demonstrated that the antibody was able to deplete CD163+ TAMs and this depletion led to an almost complete inhibition of tumor growth, which was more effective than pan-TAM blockade using CSF1R and CCR2 inhibition. The depletion of CD163+ TAMs correlated with an increase in tumor-infiltrating lymphocytes and when T cells were depleted, the effect on tumor growth restriction was reversed. This model suggests that tumor regression caused by the depletion of CD163+ TAM subsets, was driven by the recruitment and activation of lymphocytes, demonstrating the important underlying interactions between T cells and macrophage subsets within the TME: TAMs may prevent effective immune responses against melanoma by restricting T cell activation and recruitment, and by promoting an anti-inflammatory environment. Furthermore, this suggests that combining treatments which target both lymphoid and myeloid compartments of the immune system may greatly improve immunogenic cancer killing and may hold therapeutic promise. Such therapies are currently restricted to preclinical studies.

#### MARCO

Another marker of interest is the scavenger receptor MARCO, expressed on TAMs which appear to be tumor-promoting, in melanoma and other cancers.^110,111^ Although the exact pro-tumor properties of such TAMs have yet to be fully elucidated, it appears that MARCO is expressed selectively on tumor-promoting TAMs in the TME, including on perivascular TAMs with a role in angiogenesis and supporting tumor growth.^112^ One group has demonstrated, using both *in vitro* co-culture and *in vivo* models, that binding of an anti-MARCO IgG1 mAb caused target internalization and resulted in changes in the metabolic profile of immunosuppressive macrophages.^112^ These functions appeared to be dependent on the ability of the antibody to engage with the inhibitory FcγRIIb on macrophages. The TME was altered, featuring more pro-inflammatory macrophages, and treatment with anti-MARCO not only led to tumor shrinkage but also augmented the effects of anti-CTLA-4 immunotherapy through an NK cell-dependent mechanism. Sequential blocking of FcγRs demonstrated that this effect was FcγRIIb-dependent, suggesting engagement of the Fc portion of the antibody, and subsequent signaling through FcRs expressed on macrophages, is as important as creating a Fab portion which targets an antigen found only in the TME. Within the TME, MARCO appears to be solely expressed by anti-inflammatory but not pro-inflammatory macrophage subsets.^112^ However, outside of the TME, it is found on tissue resident macrophages in lung, lymph nodes, spleen, and peritoneum as well as on activated dendritic cells.^112^ This target is therefore not specific to TAMs and anti-MARCO treatments could have unwanted toxicity through impacting macrophages in healthy tissues. There are no clinical trials currently ongoing to test anti-MARCO in patients.

Despite the lack of ongoing trials of anti-MARCO mAbs, the combination of approaches, whereby both the immune system and specific cancer antigens are targeted, may prove to be a successful mechanism to target specific tumor resident macrophage subsets and to repolarize them. Engineering antibodies with Fab regions to bind cancer antigens in conjunction with Fc regions which can bind to, and ultimately change, the effector function of immune cells is an emerging field and one that holds much promise in generating an immune response which is specific to the TME.

## Engaging macrophage Fc-mediated effector functions with monoclonal antibodies

Macrophages express multiple FcRs, which, when engaged with antibodies via their Fc regions, can lead to different cellular responses, depending on the combination of FcR expression profile of the effector cells and the antibody isotype.^64,70^ This combination may have an impact on whether a pro- or antitumor response is coordinated. Macrophages are abundant in the TME of melanoma and have been shown to play essential roles in ADCC and ADCP, and in the secretion of pro-inflammatory mediators in response to FcR signaling. Designing IgG antibodies with high affinity to activatory FcRs (FcγRI, FcγRIIa, FcγRIIIa), all of which may be expressed by macrophages, or with low affinity for the inhibitory FcγRIIb, also expressed in TAMs, can help optimize antibody–macrophage interactions and may be a strategy to promote a pro-inflammatory and antitumor environment.^113–115^

IgG class mAbs currently used in clinic may have anti-cancer functions via FcRs. This is likely the case with ipilimumab, an IgG1 isotype antibody, since it has been reported that its ability to engage FcγRIIIa can lead to the cytotoxic killing of CTLA-4-expressing Tregs by effector cells such as macrophages and NK cells.^81^ In contrast, one contributor to immune evasion and melanoma progression may be the signaling through inhibitory FcRs on TAMs. For example, it has been shown that IgG4 is expressed in chronic inflammation states, and in the TME of melanoma and other solid tumors.^116,117^ IgG4 has a high affinity for FcγRIIb, which signals through a downstream ITIM domain, leading to immune cell inhibition. IgG4 isotype antibodies can interact with FcγRIIb on the surface of macrophages (see Table 1), and this may lead to impaired antitumor responses.^118,119^ Consistent with the reported immune-modulating effects of IgG4, elevated serum levels of IgG4 in patients with melanoma have been associated with poorer prognosis.^117,120^ Further work is needed to investigate how these interactions may contribute to melanoma growth and survival and may reveal new prognostic markers, and strategies to interrupt this pro-tumor signaling pathway.

On the other hand, IgE class antibodies specific for cancer-associated antigens may be able to activate macrophages. IgE antibodies which operate via a different set of activatory Fc receptors to IgG, have shown some promise in preclinical models and early clinical testing. IgE engagement of the FcεR on monocytes and macrophages has been shown to polarize these cells, including the immunoregulatory macrophage subsets, toward pro-inflammatory phenotypes and to enhance their antitumor activity.^121,122^ Importantly in the context of the TME, this involves the increased secretion of pro-inflammatory cytokines, which could change the immune milieu of the TME.^123^ It is therefore possible to influence TAMs in tumors via engineering antibodies with Fc regions of a class different to the conventional IgG.

It is also possible that TAM activation by checkpoint inhibitor antibodies could lead to depletion of pro-inflammatory immune cells. For example, TAMs engaged by anti-PD-1 antibodies could target PD-1-expressing effector T cells.^79,80^ However, engineering these antibodies with Fc regions of the IgG4 isotype, it is possible that the affinity of IgG4 for the inhibitory FcγRIIb can be utilized to reduce macrophage activation. For example, both anti-PD-1 mAbs, nivolumab and pembrolizumab, are IgG4, in contrast to ipilimumab, an IgG1 mAb, the mechanism of action of which, as discussed above, benefits from macrophage activation. In addition to this, Fc engineering of antibodies to generate agents with reduced or no binding to FcRs on TAMs is being explored, with two such anti-PD-1 inhibitors are currently in phase 1/2 clinical trials. Tislelizumab, a humanized IgG4 with minimal FcR binding is currently in phase 1/2 trials, in combination with other treatment modalities (NCT04924413, NCT04924413). Similarly, prolgolimab, an IgG1 mAb containing the Fc-silencing “LALA” mutation is being explored (NCT05120024).

Furthermore, the inhibitory FcγRIIb is specifically targeted when agonistic antibodies, such as agonistic CD40 mAbs are used.^124^ Hyper cross-linking by FcRs of anti-CD40 mAbs is required for stimulation, unlike the immune checkpoint blocking mAbs. Specifically, anti-CD40 mAbs require FcγRIIb-mediated crosslinking for efficacy.^125^ An enhanced IgG1, with a stronger binding affinity to FcγRIIb and lower affinity for FcγRIIa, has led to more potent effects of such agonistic antibodies. These findings demonstrate the importance of Fc design in antibody engineering.^126^

The efficacy of mAbs can therefore be influenced and fine-tuned by the design of Fc regions: where interactions with macrophages and the target of mAbs is detrimental, designing isotypes with a lower affinity for activatory receptors or higher affinity for inhibitory FcγRIIb may be beneficial. Where monocyte and macrophage ADCP, ADCC and inflammatory properties aid anticancer functions, engineering the Fc with high affinity for activatory FcRs could increase the efficacy of such antibodies. This may be the case for mAbs targeting cancer-antigens.

### Targeting cancer-specific antigens

mAbs provide an opportunity to target specific immune cells within the TME. By designing the Fab region to recognize a specific cancer antigen, and the Fc portion to have a high affinity for FcRs expressed on immune cells within the TME, for example macrophages, colocalization of both antigen and effector cell is enabled, enhancing the tumor cell killing functions of specific immune cells within the TME. Although this review is focused on how to target TAMs with mAbs, it is important to note that immune cells, other than monocytes and macrophages, also present within the TME, express FcRs (see Table 1). As with the “off-target” impact of checkpoint inhibitors on macrophage functions, mAbs designed to target macrophage FcRs, may be able to engage with and impact multiple cell types. Given their abundance in the TME, it is likely that TAMs will be key effector cells if FcRs are required for the antitumor functions of monoclonal antibodies recognizing cancer antigens.

A suitable target antigen for antibodies should ideally be expressed at high levels on tumor cells while not expressed or lowly expressed on non-malignant tissues. Such cancer-associated antigens can be hard to identify, since targeting cancer ultimately means targeting self. For example, while over-expressed by 15–20% of breast cancers, HER2 is also expressed by gastrointestinal, respiratory, reproductive, and urinary tract cells as well as in the skin, breast and placenta.^127^ Its expression on cardiomyocytes is thought to be the underlying pathogenic mechanism of cardiac side effects seen in patients treated with the anti-HER2 antibody trastuzumab.^128^

### Phosphatidylserine

Although not restricted exclusively to tumor cells, phosphatidylserine (PS) is a target currently being explored in clinical trials in cancer types other than melanoma (in head and neck cancer, NCT04150900; hepatocellular cancer, NCT03519997, NCT05249569; and glioblastoma, NCT03139916). PS is expressed on the inner leaflet of the plasma membrane in healthy cells. However, it is transported to the cell surface in apoptotic cells. Once bound to its receptor on macrophages, efferocytosis is triggered, allowing the clearing of dead and dying cells.^129^ The process is immunoregulatory, contributing to inflammation resolution, with efferocytosis causing macrophages to become more immunosuppressive and attenuate NK cell and DC cell activation.^130^ Aside from being expressed on apoptotic cells, PS expression is upregulated on the cell surface of tumor cells, including in melanoma. Antibodies blocking PS have demonstrated antitumor activity by reducing the immunosuppressive effects caused by efferocytosis, increasing immune cell activation, destruction of tumor vasculature and promotion of a more pro-inflammatory TME.^130,131^

In melanoma, exploration have been restricted to preclinical studies. In a mouse model of melanoma, a mouse IgG2 equivalent of bavituximab, mch1N11, was combined with radiotherapy and anti-PD1 immune checkpoint inhibitor.^129^ The combination delayed tumor growth and prolonged survival of mice compared to treatment without bavituximab. The study also demonstrated an increase in TAM infiltration, which had a more pro-inflammatory phenotype (CD206- MHCII+), and increased secretion of pro-inflammatory cytokines in the TME.

The mechanism of action of bavituximab demonstrates the proof of concept that using mAbs against a cancer-antigen can enhance the FcR effects of local immune cells. However, PS is not restricted just to the TME of melanoma. It is expressed by platelets, myeloid cells, activated B and T cells and DCs, and although toxicity has not been reported in studies in other cancer types,^132^ the potential for off-target effects of bavituximab warrants further investigation.

### Chondroitin sulfate proteoglycan 4

Chondroitin sulfate proteoglycan 4 (CSPG4) is a cancer antigen which may be more selective for cancer cells. CSPG4 is expressed in 70% of the melanomas with a low and restricted expression profile in healthy tissue.^133^ Monoclonal antibodies designed against this target which can engage the FcRs on immune cells, including monocytes and macrophages, are under development in preclinical models. An anti-CSPG4 IgG1 monoclonal antibody has been reported to increase macrophage recruitment in a fully humanized mouse model of melanoma,^118^ and repeated administration of an anti-CSPG4 IgE monoclonal antibody therapy is well tolerated in immunocompetent animal models.^133^ The exact effect of anti-CSPG4 mAbs on macrophages remains unknown but warrants exploration, since polarizing TAMs and enhancing their antitumor properties may be a potential mechanism by which anti-CSPG4 can exert therapeutic effects.

## Conclusion

Given their key roles in melanoma growth and survival, tumor-associated macrophages may be a promising target for an array of novel immunotherapeutic strategies. Their plasticity means these cells can be manipulated, both by their environment, for example factors within the TME, and by exogenous stimuli, such as therapeutic immunoglobulins. Many promising ways to clinically target macrophages with mAbs are being explored. The Fab portion of mAbs can be used to block signaling pathways which promote melanoma growth and survival, for example blocking TAM chemoattractant pathways, blocking immunoregulatory axes such as SIPRα/CD47 and inhibiting immunoregulatory MIF. Given the multiple ongoing trials, using agonistic mAbs, such as CD40 and TLR agonists, to promote cancer-cell killing by immune cells holds much promise. The Fab portion can also target markers of immunoregulatory TAM subsets, such as CD163, which may prove to be a promising candidate for antibody–drug conjugates (ADC). The design of Fc portions may prove to be just as important: intelligent design may lead to enhanced efficacy of mAb therapies, by carefully considering target expression distribution and ensuring effective or no engagement with immune cells through the binding of FcRs, as required. For example, by designing anti-cancer antigen specific Fab regions, mAbs can penetrate and be crosslinked at the tumor site in the presence of tumor antigen-expressing cells, i.e., by macrophages, within the TME, where an enhanced immune response is needed. By designing an antibody’s Fc region to optimally bind to an activatory FcR which generates a pro-inflammatory response in macrophages, mAbs can not only influence the effector function of macrophages but could also alter the cytokine milieu of the TME to enhance anticancer activity.

## Supplementary Material

Supplemental Material

## References

[1] Euromelanoma. 2020 Melanoma skin cancer report. cited 2021 November 15. Available from: https://www.melanomauk.org.uk/2020-melanoma-skin-cancer-report

[2] CRUK. Melanoma skin cancer statistics, cancer research UK February. 2021. Available from: https://www.cancerresearchuk.org/health-professional/cancer-statistics/statistics-by-cancer-type/melanoma-skin-cancer. Accessed 25 September 2022.

[3] Farkona S, Diamandis EP, Blasutig IM. Cancer immunotherapy: the beginning of the end of cancer? BMC Med. 2016;14(1):73. doi:10.1186/s12916-016-0623-5. 27151159 PMC4858828

[4] Rodríguez-Cerdeira C, Carnero Gregorio M, López-Barcenas A, Sánchez-Blanco E, Sánchez-Blanco B, Fabbrocini G, Bardhi B, Sinani A, Guzman RA. Advances in immunotherapy for melanoma: a comprehensive review. Mediators Inflamm. 2017;2017:3264217. doi:10.1155/2017/3264217. 28848246 PMC5564072

[5] Moreira A, Heinzerling L, Bhardwaj N, Friedlander P. Current melanoma treatments: where do we stand? Cancers (Basel). 2021;13(2):221. doi:10.3390/cancers13020221. PMC7827568 33435389

[6] Savoia P, Zavattaro E, Cremona O. Clinical Implications of acquired BRAF inhibitors resistance in melanoma. Int J Mol Sci. 2020;21(24):9730. doi:10.3390/ijms21249730. PMC7766699 33419275

[7] Wolchok JD, Chiarion-Sileni V, Gonzalez R, Rutkowski P, Grob -J-J, Cowey CL, Lao CD, Wagstaff J, Schadendorf D, Ferrucci PF. Overall survival with combined nivolumab and ipilimumab in advanced melanoma. N Engl J Med. 2017;377(14):1345–16. doi:10.1056/NEJMoa1709684. 28889792 PMC5706778

[8] Ramos-Casals M, Brahmer JR, Callahan MK, Flores-Chávez A, Keegan N, Khamashta MA, Lambotte O, Mariette X, Prat A, Suárez-Almazor ME. Immune-related adverse events of checkpoint inhibitors. Nat Rev Dis Primers. 2020;6(1):38. doi:10.1038/s41572-020-0160-6. 32382051 PMC9728094

[9] Iacono D, Basile D, Gerratana L, Vitale MG, Pelizzari G, Cinausero M, Poletto E, Puglisi F, Fasola G, Minisini AM, et al. Prognostic role of disease extent and lymphocyte-monocyte ratio in advanced melanoma. Melanoma Res. 2019;29(5):510–515. doi:10.1097/CMR.0000000000000584. 30702508

[10] Zhang QW, Liu L, Gong CY, Shi H-S, Zeng Y-H, Wang X-Z, Zhao Y-W, Wei Y-Q. Prognostic significance of tumor-associated macrophages in solid tumor: a meta-analysis of the literature. PLoS One. 2012;7(12):e50946. doi:10.1371/journal.pone.0050946. 23284651 PMC3532403

[11] López-Janeiro Á, Padilla-Ansala C, de Andrea CE, Hardisson D, Melero I. Prognostic value of macrophage polarization markers in epithelial neoplasms and melanoma. A systematic review and meta-analysis. Mod Pathol. 2020;33(8):1458–1465. doi:10.1038/s41379-020-0534-z. 32291396

[12] Falleni M, Savi F, Tosi D, Agape E, Cerri A, Moneghini L, Bulfamante GP. M1 and M2 macrophages’ clinicopathological significance in cutaneous melanoma. Melanoma Res. 2017;27(3):200–210. doi:10.1097/CMR.0000000000000352. 28272106

[13] Shapouri‐Moghaddam A, Mohammadian S, Vazini H, Taghadosi M, Esmaeili S-A, Mardani F, Seifi B, Mohammadi A, Afshari JT, Sahebkar A. Macrophage plasticity, polarization, and function in health and disease. J Cell Physiol. 2018;233(9):6425–6440. doi:10.1002/jcp.26429. PMC13482560 29319160

[14] Murray PJ. Macrophage polarization. Annu Rev Physiol. 2017;79 1:541–566. doi:10.1146/annurev-physiol-022516-034339. 27813830

[15] Tan Y, Wang M, Zhang Y, Ge S, Zhong F, Xia G, Sun C. Tumor-associated macrophages: a potential target for cancer therapy. Front Oncol. 2021;11:693517. doi:10.3389/fonc.2021.693517. 34178692 PMC8222665

[16] Mantovani A, Marchesi F, Malesci A, Laghi L, Allavena P. Tumour-associated macrophages as treatment targets in oncology. Nat Rev Clin Oncol. 2017;14(7):399–416. doi:10.1038/nrclinonc.2016.217. 28117416 PMC5480600

[17] Zhou J, Tang Z, Gao S, Li C, Feng Y, Zhou X. Tumor-associated macrophages: recent insights and therapies. Front Oncol. 2020;10:188. doi:10.3389/fonc.2020.00188. 32161718 PMC7052362

[18] Yang L, Zhang Y. Tumor-associated macrophages: from basic research to clinical application. J Hematol Oncol. 2017;10(1):58. doi:10.1186/s13045-017-0430-2. 28241846 PMC5329931

[19] Marconi C, Bianchini F, Mannini A, Mugnai G, Ruggieri S, Calorini L. Tumoral and macrophage uPAR and MMP-9 contribute to the invasiveness of B16 murine melanoma cells. Clin Exp Metastasis. 2008;25(3):225–231. doi:10.1007/s10585-007-9136-0. 18071911

[20] Rőszer T. Understanding the mysterious M2 macrophage through activation markers and effector mechanisms. Mediators Inflamm. 2015;2015:816460. doi:10.1155/2015/816460. 26089604 PMC4452191

[21] Murray PJ, Allen JE, Biswas SK, Fisher E, Gilroy D, Goerdt S, Gordon S, Hamilton J, Ivashkiv L, Lawrence T. Macrophage activation and polarization: nomenclature and experimental guidelines. Immunity. 2014;41(1):14–20. doi:10.1016/j.immuni.2014.06.008. 25035950 PMC4123412

[22] Azizi E, Carr AJ, Plitas G, Cornish AE, Konopacki C, Prabhakaran S, Nainys J, Wu K, Kiseliovas V, Setty M. Single-cell map of diverse immune phenotypes in the breast tumor microenvironment. Cell. 2018;174(5):1293–1308.e36. doi:10.1016/j.cell.2018.05.060. 29961579 PMC6348010

[23] Scali E, Mignogna C, Di Vito A, Presta I, Camastra C, Donato G, Bottoni U. Inflammation and macrophage polarization in cutaneous melanoma: histopathological and immunohistochemical study. Int J Immunopathol Pharmacol. 2016;29(4):715–719. doi:10.1177/0394632016650895. 27387897 PMC5806828

[24] Rosell AP, Maiques O, Chakravarty P, Ombrato L, Sanz-Moreno V, Malanchi I. Early functional mismatch between breast cancer cells and their tumour microenvironment suppresses long term growth. Cancer Letters. 2021;544:215800. 10.1016/j.canlet.2022.215800. bioRxiv. 2021:2021.06.15.448466 35803476

[25] Hashimoto D, Chow A, Noizat C, Teo P, Beasley M, Leboeuf M, Becker C, See P, Price J, Lucas D. Tissue-resident macrophages self-maintain locally throughout adult life with minimal contribution from circulating monocytes. Immunity. 2013;38(4):792–804. doi:10.1016/j.immuni.2013.04.004. 23601688 PMC3853406

[26] Nesbit M, Schaider H, Miller TH, Herlyn M. Low-level monocyte chemoattractant protein-1 stimulation of monocytes leads to tumor formation in nontumorigenic melanoma cells. J Immunol. 2001;166(11):6483–6490. doi:10.4049/jimmunol.166.11.6483. 11359798

[27] Cortez-Retamozo V, Etzrodt M, Newton A, Rauch PJ, Chudnovskiy A, Berger C, Ryan RJH, Iwamoto Y, Marinelli B, Gorbatov R. Origins of tumor-associated macrophages and neutrophils. Proc Natl Acad Sci U S A. 2012;109(7):2491–2496. doi:10.1073/pnas.1113744109. 22308361 PMC3289379

[28] Adams R, Moser B, Karagiannis SN, Lacy KE. Chemokine pathways in cutaneous melanoma: their modulation by cancer and exploitation by the clinician. Cancers (Basel). 2021;13(22):5625. doi:10.3390/cancers13225625. 34830780 PMC8615762

[29] Laviron M, Boissonnas A. Ontogeny of tumor-associated macrophages. Front Immunol. 2019;10:1799. doi:10.3389/fimmu.2019.01799. 31417566 PMC6684758

[30] Christofides A, Strauss L, Yeo A, Cao C, Charest A, Boussiotis VA. The complex role of tumor-infiltrating macrophages. Nat Immunol. 2022;23(8):1148–1156. doi:10.1038/s41590-022-01267-2. 35879449 PMC10754321

[31] Lahmar Q, Keirsse J, Laoui D, Movahedi K, Van Overmeire E, Van Ginderachter JA. Tissue-resident versus monocyte-derived macrophages in the tumor microenvironment. Biochim Biophys Acta. 2016;1865(1):23–34. doi:10.1016/j.bbcan.2015.06.009. 26145884

[32] Georgouli M, Herraiz C, Crosas-Molist E, Fanshawe B, Maiques O, Perdrix A, Pandya P, Rodriguez-Hernandez I, Ilieva KM, Cantelli G. Regional activation of myosin II in cancer cells drives tumor progression via a secretory cross-talk with the immune microenvironment. Cell. 2019;176(4):757–774 e23. doi:10.1016/j.cell.2018.12.038. 30712866 PMC6370915

[33] Wang T, Ge Y, Xiao M, Lopez-Coral A, Azuma R, Somasundaram R, Zhang G, Wei Z, Xu X, Rauscher FJ, et al. Melanoma-derived conditioned media efficiently induce the differentiation of monocytes to macrophages that display a highly invasive gene signature. Pigment Cell Melanoma Res. 2012;25(4):493–505. doi:10.1111/j.1755-148X.2012.01005.x. 22498258 PMC3615702

[34] Wenes M, Shang M, Di Matteo M, Goveia J, Martín-Pérez R, Serneels J, Prenen H, Ghesquière B, Carmeliet P, Mazzone M, et al. Macrophage metabolism controls tumor blood vessel morphogenesis and metastasis. Cell Metab. 2016;24(5):701–715. doi:10.1016/j.cmet.2016.09.008. 27773694

[35] Li S, Yu J, Huber A, Kryczek I, Wang Z, Jiang L, Li X, Du W, Li G, Wei S, et al. Metabolism drives macrophage heterogeneity in the tumor microenvironment. Cell Rep. 2022;39(1):110609. doi:10.1016/j.celrep.2022.110609. 35385733 PMC9052943

[36] Cheng S, Li Z, Gao R, Xing B, Gao Y, Yang Y, Qin S, Zhang L, Ouyang H, Du P, et al. A pan-cancer single-cell transcriptional atlas of tumor infiltrating myeloid cells. Cell. 2021;184(3):792–809.e23. doi:10.1016/j.cell.2021.01.010. 33545035

[37] Mulder K, Patel AA, Kong WT, Piot C, Halitzki E, Dunsmore G, Khalilnezhad S, Irac SE, Dubuisson A, Chevrier M, et al. Cross-tissue single-cell landscape of human monocytes and macrophages in health and disease. Immunity. 2021;54(8):1883–1900.e5. doi:10.1016/j.immuni.2021.07.007. 34331874

[38] Ma RY, Black A, Qian BZ. Macrophage diversity in cancer revisited in the era of single-cell omics. Trends Immunol. 2022;43(7):546–563. doi:10.1016/j.it.2022.04.008. 35690521

[39] Korbecki J, Kojder K, Simińska D, Bohatyrewicz R, Gutowska I, Chlubek D, Baranowska-Bosiacka I. CC chemokines in a tumor: a review of pro-cancer and anti-cancer properties of the ligands of receptors CCR1, CCR2, CCR3, and CCR4. Int J Mol Sci. 2020;21(21):8412. 10.3390/ijms21218412 PMC7665155 33182504

[40] Samaniego R, Gutiérrez-González A, Gutiérrez-Seijo A, Sánchez-Gregorio S, García-Giménez J, Mercader E, Márquez-Rodas I, Avilés JA, Relloso M, Sánchez-Mateos P. CCL20 expression by tumor-associated macrophages predicts progression of human primary cutaneous melanoma. Cancer Immunol Res. 2018;6(3):267–275. doi:10.1158/2326-6066.CIR-17-0198. 29362221

[41] Kakizaki A, Fujimura T, Furudate S, Kambayashi Y, Yamauchi T, Yagita H, Aiba S. Immunomodulatory effect of peritumorally administered interferon-beta on melanoma through tumor-associated macrophages. Oncoimmunology. 2015;4(11):e1047584. doi:10.1080/2162402X.2015.1047584. 26451326 PMC4589056

[42] Naama HA, Mack VE, Smyth GP, Stapleton PP, Daly JM. Macrophage effector mechanisms in melanoma in an experimental study. Arch Surg. 2001;136(7):804–809. doi:10.1001/archsurg.136.7.804. 11448395

[43] Tang M, Wang Y, Han S, Guo S, Xu N, Guo J. Endogenous PGE(2) induces MCP-1 expression via EP4/p38 MAPK signaling in melanoma. Oncol Lett. 2013;5(2):645–650. doi:10.3892/ol.2012.1047. 23420676 PMC3573118

[44] Allavena P, Piemonti L, Longoni D, Bernasconi S, Stoppacciaro A, Ruco L, Mantovani A. IL-10 prevents the differentiation of monocytes to dendritic cells but promotes their maturation to macrophages. Eur J Immunol. 1998;28(1):359–369. 9485215 10.1002/(SICI)1521-4141(199801)28:01<359::AID-IMMU359>3.0.CO;2-4

[45] Michielon E, López González M, Burm JLA, Waaijman T, Jordanova ES, de Gruijl TD, Gibbs S. Micro-environmental cross-talk in an organotypic human melanoma-in-skin model directs M2-like monocyte differentiation via IL-10. Cancer Immunol Immunother. 2020;69(11):2319–2331. doi:10.1007/s00262-020-02626-4. 32507967 PMC7568725

[46] Mantovani A, Sozzani S, Locati M, Allavena P, Sica A. Macrophage polarization: tumor-associated macrophages as a paradigm for polarized M2 mononuclear phagocytes. Trends Immunol. 2002;23(11):549–555. doi:10.1016/S1471-4906(02)02302-5. 12401408

[47] Gordon SR, Maute RL, Dulken BW, Hutter G, George BM, McCracken MN, Gupta R, Tsai JM, Sinha R, Corey D, et al. PD-1 expression by tumour-associated macrophages inhibits phagocytosis and tumour immunity. Nature. 2017;545(7655):495–499. doi:10.1038/nature22396. 28514441 PMC5931375

[48] Soumoy L, Kindt N, Ghanem G, Saussez S, Journe F. Role of Macrophage migration inhibitory factor (MIF) in melanoma. Cancers (Basel). 2019;11(4):529. doi:10.3390/cancers11040529. PMC6520935 31013837

[49] Yaddanapudi K, Putty K, Rendon BE, Lamont GJ, Faughn JD, Satoskar A, Lasnik A, Eaton JW, Mitchell RA. Control of tumor-associated macrophage alternative activation by macrophage migration inhibitory factor. J Immunol. 2013;190(6):2984–2993. doi:10.4049/jimmunol.1201650. 23390297 PMC3593945

[50] Figueiredo CR, Azevedo RA, Mousdell S, Resende-Lara PT, Ireland L, Santos A, Girola N, Cunha RLOR, Schmid MC, Polonelli L. Blockade of MIF-CD74 signalling on macrophages and dendritic cells restores the antitumour immune response against metastatic melanoma. Front Immunol. 2018;9:1132. doi:10.3389/fimmu.2018.01132. 29875777 PMC5974174

[51] de Azevedo RA, Shoshan E, Whang S, Markel G, Jaiswal AR, Liu A, Curran MA, Travassos LR, Bar-Eli M. MIF inhibition as a strategy for overcoming resistance to immune checkpoint blockade therapy in melanoma. Oncoimmunology. 2020;9(1):1846915. doi:10.1080/2162402X.2020.1846915. 33344042 PMC7733907

[52] Wang T, Xiao M, Ge Y, Krepler C, Belser E, Lopez-Coral A, Xu X, Zhang G, Azuma R, Liu Q. BRAF inhibition stimulates melanoma-associated macrophages to drive tumor growth. Clin Cancer Res. 2015;21(7):1652–1664. doi:10.1158/1078-0432.CCR-14-1554. 25617424 PMC4383683

[53] Torisu H, Ono M, Kiryu H, Furue M, Ohmoto Y, Nakayama J, Nishioka Y, Sone S, Kuwano M. Macrophage infiltration correlates with tumor stage and angiogenesis in human malignant melanoma: possible involvement of TNFalpha and IL-1alpha. Int J Cancer. 2000;85(2):182–188. doi:10.1002/(SICI)1097-0215(20000115)85:2<182::AID-IJC6>3.0.CO;2-M. 10629075

[54] Jensen TO, Schmidt H, Møller HJ, Høyer M, Maniecki MB, Sjoegren P, Christensen IJ, Steiniche T. Macrophage markers in serum and tumor have prognostic impact in American joint committee on cancer stage I/II melanoma. J Clin Oncol. 2009;27(20):3330–3337. doi:10.1200/JCO.2008.19.9919. 19528371

[55] Salmi S, Siiskonen H, Sironen R, Tyynelä-Korhonen K, Hirschovits-Gerz B, Valkonen M, Auvinen P, Pasonen-Seppänen S. The number and localization of CD68+ and CD163+ macrophages in different stages of cutaneous melanoma. Melanoma Res. 2019;29(3):237–247. doi:10.1097/CMR.0000000000000522. 30399061 PMC6493694

[56] Tian Y, Guo Y, Zhu P, Zhang D, Liu S, Tang M, Wang Y, Jin Z, Li D, Yan D. TRIM59 loss in M2 macrophages promotes melanoma migration and invasion by upregulating MMP-9 and Madcam1. Aging (Albany NY). 2019;11(19):8623–8641. doi:10.18632/aging.102351. 31600735 PMC6814609

[57] Fu LQ, Du WL, Cai MH, Yao J-Y, Zhao -Y-Y, Mou X-Z. The roles of tumor-associated macrophages in tumor angiogenesis and metastasis. Cell Immunol. 2020;353:104119. doi:10.1016/j.cellimm.2020.104119. 32446032

[58] Qian BZ, Pollard JW. Macrophage diversity enhances tumor progression and metastasis. Cell. 2010;141(1):39–51. doi:10.1016/j.cell.2010.03.014. 20371344 PMC4994190

[59] Qian B, Deng Y, Im JH. A distinct macrophage population mediates metastatic breast cancer cell extravasation, establishment and growth. PLoS One. 2009;4(8):e6562. doi:10.1371/journal.pone.0006562. 19668347 PMC2721818

[60] Joyce JA, Pollard JW. Microenvironmental regulation of metastasis. Nat Rev Cancer. 2009;9(4):239–252. doi:10.1038/nrc2618. 19279573 PMC3251309

[61] Kaplan RN, Riba RD, Zacharoulis S, Bramley AH, Vincent L, Costa C, MacDonald DD, Jin DK, Shido K, Kerns SA, et al. VEGFR1-positive haematopoietic bone marrow progenitors initiate the pre-metastatic niche. Nature. 2005;438(7069):820–827. doi:10.1038/nature04186. 16341007 PMC2945882

[62] Orgaz JL, Crosas-Molist E, Sadok A, Perdrix-Rosell A, Maiques O, Rodriguez-Hernandez I, Monger J, Mele S, Georgouli M, Bridgeman V, et al. Myosin II reactivation and cytoskeletal remodeling as a hallmark and a vulnerability in melanoma therapy resistance. Cancer Cell. 2020;37(1):85–103.e9. doi:10.1016/j.ccell.2019.12.003. 31935375 PMC6958528

[63] Bournazos S, Wang TT, Dahan R, Maamary J, Ravetch JV. Signaling by antibodies: recent progress. Annu Rev Immunol. 2017;35 1:285–311. doi:10.1146/annurev-immunol-051116-052433. 28446061 PMC5613280

[64] Bianchini R, Karagiannis SN, Jordakieva G, Jensen-Jarolim E. The role of IgG4 in the fine tuning of tolerance in IgE-mediated allergy and cancer. Int J Mol Sci. 2020;21(14):5017. doi:10.3390/ijms21145017. PMC7404042 32708690

[65] Weiner LM, Surana R, Wang S. Monoclonal antibodies: versatile platforms for cancer immunotherapy. Nat Rev Immunol. 2010;10(5):317–327. doi:10.1038/nri2744. 20414205 PMC3508064

[66] Bournazos S, Gupta A, Ravetch JV. The role of IgG Fc receptors in antibody-dependent enhancement. Nat Rev Immunol. 2020;20(10):633–643. doi:10.1038/s41577-020-00410-0. 32782358 PMC7418887

[67] Bruhns P, Jönsson F. Mouse and human FcR effector functions. Immunol Rev. 2015;268(1):25–51. doi:10.1111/imr.12350. 26497511

[68] Bruhns P, Iannascoli B, England P, Mancardi DA, Fernandez N, Jorieux S, Daëron M. Specificity and affinity of human Fc gamma receptors and their polymorphic variants for human IgG subclasses. Blood. 2009;113(16):3716–3725. doi:10.1182/blood-2008-09-179754. 19018092

[69] Hogarth PM, Pietersz GA. Fc receptor-targeted therapies for the treatment of inflammation, cancer and beyond. Nat Rev Drug Discov. 2012;11(4):311–331. doi:10.1038/nrd2909. 22460124

[70] Chenoweth AM, Wines BD, Anania JC, Mark Hogarth P. Harnessing the immune system via FcγR function in immune therapy: a pathway to next-gen mAbs. Immunol Cell Biol. 2020;98(4):287–304. doi:10.1111/imcb.12326. 32157732 PMC7228307

[71] Francisco JA, Cerveny CG, Meyer DL, Mixan BJ, Klussman K, Chace DF, Rejniak SX, Gordon KA, DeBlanc R, Toki BE. cAC10-vcMMAE, an anti-CD30-monomethyl auristatin E conjugate with potent and selective antitumor activity. Blood. 2003;102(4):1458–1465. doi:10.1182/blood-2003-01-0039. 12714494

[72] Cameron D, Piccart-Gebhart MJ, Gelber RD, Procter M, Goldhirsch A, de Azambuja E, Castro G, Untch M, Smith I, Gianni L, et al. 11 years’ follow-up of trastuzumab after adjuvant chemotherapy in HER2-positive early breast cancer: final analysis of the HERceptin adjuvant (HERA) trial. Lancet. 2017;389(10075):1195–1205. doi:10.1016/S0140-6736(16)32616-2. 28215665 PMC5465633

[73] Zahavi D, Weiner L. Monoclonal antibodies in cancer therapy. Antibodies (Basel). 2020;9(3). doi:10.3390/antib9030034. PMC7551545 32698317

[74] Willsmore ZN, Coumbe BGT, Crescioli S, Reci S, Gupta A, Harris RJ, Chenoweth A, Chauhan J, Bax HJ, McCraw A, et al. Combined anti-PD-1 and anti-CTLA-4 checkpoint blockade: treatment of melanoma and immune mechanisms of action. Eur J Immunol. 2021;51(3):544–556. doi:10.1002/eji.202048747. 33450785

[75] Luke JJ, Flaherty KT, Ribas A, Long GV. Targeted agents and immunotherapies: optimizing outcomes in melanoma. Nat Rev Clin Oncol. 2017;14(8):463–482. doi:10.1038/nrclinonc.2017.43. 28374786

[76] Hartley GP, Chow L, Ammons DT, Wheat WH, Dow SW. Programmed cell death ligand 1 (PD-L1) signaling regulates macrophage proliferation and activation. Cancer Immunol Res. 2018;6(10):1260–1273. doi:10.1158/2326-6066.CIR-17-0537. 30012633

[77] Sahni S, Valecha G, Sahni A. Role of Anti-PD-1 antibodies in advanced melanoma: the era of immunotherapy. Cureus. 2018;10(12):e3700. doi:10.7759/cureus.3700. 30788189 PMC6372252

[78] Neubert NJ, Schmittnaegel M, Bordry N, Nassiri S, Wald N, Martignier C, Tillé L, Homicsko K, Damsky W, Maby-El Hajjami H. T cell-induced CSF1 promotes melanoma resistance to PD1 blockade. Sci Transl Med. 2018;10(436). doi:10.1126/scitranslmed.aan3311. PMC5957531 29643229

[79] Arlauckas SP, Garris CS, Kohler RH, Kitaoka M, Cuccarese MF, Yang KS, Miller MA, Carlson JC, Freeman GJ, Anthony RM, et al. In vivo imaging reveals a tumor-associated macrophage-mediated resistance pathway in anti-PD-1 therapy. Sci Transl Med. 2017;9(389). doi:10.1126/scitranslmed.aal3604. PMC5734617 28490665

[80] Zhang T, Song X, Xu L, Ma J, Zhang Y, Gong W, Zhang Y, Zhou X, Wang Z, Wang Y, et al. The binding of an anti-PD-1 antibody to FcγRΙ has a profound impact on its biological functions. Cancer Immunol Immunother. 2018;67(7):1079–1090. doi:10.1007/s00262-018-2160-x. 29687231 PMC6006217

[81] Romano E, Kusio-Kobialka M, Foukas PG, Baumgaertner P, Meyer C, Ballabeni P, Michielin O, Weide B, Romero P, Speiser DE, et al. Ipilimumab-dependent cell-mediated cytotoxicity of regulatory T cells ex vivo by nonclassical monocytes in melanoma patients. Proc Natl Acad Sci U S A. 2015;112(19):6140–6145. doi:10.1073/pnas.1417320112. 25918390 PMC4434760

[82] Simpson TR, Li F, Montalvo-Ortiz W, Sepulveda MA, Bergerhoff K, Arce F, Roddie C, Henry JY, Yagita H, Wolchok JD, et al. Fc-dependent depletion of tumor-infiltrating regulatory T cells co-defines the efficacy of anti-CTLA-4 therapy against melanoma. J Exp Med. 2013;210(9):1695–1710. doi:10.1084/jem.20130579. 23897981 PMC3754863

[83] Atzori MG, Ceci C, Ruffini F, Trapani M, Barbaccia ML, Tentori L, D’Atri S, Lacal PM, Graziani G. Role of VEGFR-1 in melanoma acquired resistance to the BRAF inhibitor vemurafenib. J Cell Mol Med. 2020;24(1):465–475. doi:10.1111/jcmm.14755. 31758648 PMC6933379

[84] Mok S, Tsoi J, Koya RC, Hu-Lieskovan S, West BL, Bollag G, Graeber TG, Ribas A. Inhibition of colony stimulating factor-1 receptor improves antitumor efficacy of BRAF inhibition. BMC Cancer. 2015;15(1):356. doi:10.1186/s12885-015-1377-8. 25939769 PMC4432503

[85] Mok S, Koya RC, Tsui C. Inhibition of CSF-1 receptor improves the antitumor efficacy of adoptive cell transfer immunotherapy. Cancer Res. 2014;74(1):153–161. doi:10.1158/0008-5472.CAN-13-1816. 24247719 PMC3947337

[86] Clinicaltrials.gov. Current clinical trials of emactuzumab. 2021. Available from: https://clinicaltrials.gov/ct2/results?term=emactuzumab&cond=Cancer&Search=Apply&age_v=&gndr=&type=&rslt=. Accessed 25 September 2022

[87] Gomez-Roca CA, Italiano A, Le Tourneau C, Cassier PA, Toulmonde M, D’angelo SP, Campone M, Weber KL, Loirat D, Cannarile MA, Jegg AM. Phase I study of emactuzumab single agent or in combination with paclitaxel in patients with advanced/metastatic solid tumors reveals depletion of immunosuppressive M2-like macrophages. Ann Oncol. 2019;30(8):1381–1392. doi:10.1093/annonc/mdz163. 31114846 PMC8887589

[88] Cassetta L, Pollard JW. Targeting macrophages: therapeutic approaches in cancer. Nat Rev Drug Discov. 2018;17(12):887–904. doi:10.1038/nrd.2018.169. 30361552

[89] Sandhu SK, Papadopoulos K, Fong PC, Patnaik A, Messiou C, Olmos D, Wang G, Tromp BJ, Puchalski TA, Balkwill F, et al. A first-in-human, first-in-class, phase I study of carlumab (CNTO 888), a human monoclonal antibody against CC-chemokine ligand 2 in patients with solid tumors. Cancer Chemother Pharmacol. 2013;71(4):1041–1050. doi:10.1007/s00280-013-2099-8. 23385782

[90] Brana I, Calles A, LoRusso PM, Yee LK, Puchalski TA, Seetharam S, Zhong B, de Boer CJ, Tabernero J, Calvo E, et al. Carlumab, an anti-C-C chemokine ligand 2 monoclonal antibody, in combination with four chemotherapy regimens for the treatment of patients with solid tumors: an open-label, multicenter phase 1b study. Target Oncol. 2015;10(1):111–123. doi:10.1007/s11523-014-0320-2. 24928772

[91] Tu MM, Abdel-Hafiz HA, Jones RT, Jean A, Hoff KJ, Duex JE, Chauca-Diaz A, Costello JC, Dancik GM, Tamburini BAJ, et al. Inhibition of the CCL2 receptor, CCR2, enhances tumor response to immune checkpoint therapy. Commun Biol. 2020;3(1):720. doi:10.1038/s42003-020-01441-y. 33247183 PMC7699641

[92] Kaur S, Cicalese KV, Bannerjee R, Roberts DD. Preclinical and clinical development of therapeutic antibodies targeting functions of CD47 in the tumor microenvironment. Antib Ther. 2020;3(3):179–192. doi:10.1093/abt/tbaa017. 33244513 PMC7687918

[93] Jalil AR, Andrechak JC, Discher DE. Macrophage checkpoint blockade: results from initial clinical trials, binding analyses, and CD47-SIRPα structure-function. Antib Ther. 2020;3(2):80–94. doi:10.1093/abt/tbaa006. 32421049 PMC7206415

[94] Kuo TC, Chen A, Harrabi O, Sockolosky JT, Zhang A, Sangalang E, Doyle LV, Kauder SE, Fontaine D, Bollini S. Targeting the myeloid checkpoint receptor SIRPα potentiates innate and adaptive immune responses to promote anti-tumor activity. J Hematol Oncol. 2020;13(1):160. doi:10.1186/s13045-020-00989-w. 33256806 PMC7706287

[95] Liu J, Xavy S, Mihardja S, Chen S, Sompalli K, Feng D, Choi T, Agoram B, Majeti R, Weissman IL, et al. Targeting macrophage checkpoint inhibitor SIRPα for anticancer therapy. JCI Insight. 2020;5(12). doi:10.1172/jci.insight.134728. PMC7406266 32427583

[96] Li DK, Wang W. Characteristics and clinical trial results of agonistic anti-CD40 antibodies in the treatment of malignancies. Oncol Lett. 2020;20(5):176. doi:10.3892/ol.2020.12037. 32934743 PMC7471753

[97] Vonderheide RH, Flaherty KT, Khalil M, Stumacher MS, Bajor DL, Hutnick NA, Sullivan P, Mahany JJ, Gallagher M, Kramer A, et al. Clinical activity and immune modulation in cancer patients treated with CP-870,893, a novel CD40 agonist monoclonal antibody. J Clin Oncol. 2007;25(7):876–883. doi:10.1200/JCO.2006.08.3311. 17327609

[98] Djureinovic D, Wang M, Kluger HM. Agonistic CD40 antibodies in cancer treatment. Cancers (Basel). 2021;13(6):1302. doi:10.3390/cancers13061302. 33804039 PMC8000216

[99] Bajor DL, Mick R, Riese MJ, Huang AC, Sullivan B, Richman LP, Torigian DA, George SM, Stelekati E, Chen F, Melenhorst JJ. Long-term outcomes of a phase I study of agonist CD40 antibody and CTLA-4 blockade in patients with metastatic melanoma. Oncoimmunology. 2018;7(10):e1468956. doi:10.1080/2162402X.2018.1468956. 30288340 PMC6169575

[100] Vonderheide RH. CD40 agonist antibodies in cancer immunotherapy. Annu Rev Med. 2020;71(1):47–58. doi:10.1146/annurev-med-062518-045435. 31412220

[101] Hoves S, Ooi CH, Wolter C, Sade H, Bissinger S, Schmittnaegel M, Ast O, Giusti AM, Wartha K, Runza V, Xu W. Rapid activation of tumor-associated macrophages boosts preexisting tumor immunity. J Exp Med. 2018;215(3):859–876. doi:10.1084/jem.20171440. 29436396 PMC5839760

[102] Anderson NR, Minutolo NG, Gill S, Klichinsky M. Macrophage-based approaches for cancer immunotherapy. Cancer Res. 2021;81(5):1201–1208. doi:10.1158/0008-5472.CAN-20-2990. 33203697

[103] Smirnov D, Schmidt JJ, Capecchi JT, Wightman PD. Vaccine adjuvant activity of 3M-052: an imidazoquinoline designed for local activity without systemic cytokine induction. Vaccine. 2011;29(33):5434–5442. doi:10.1016/j.vaccine.2011.05.061. 21641953

[104] Benonisson H, Sow HS, Breukel C, Claassens J, Brouwers C, Linssen MM, Fransen MF, Sluijter M, Ossendorp F, van Hall T. High FcγR expression on intratumoral macrophages enhances tumor-targeting antibody therapy. J Immunol. 2018;201(12):3741–3749. doi:10.4049/jimmunol.1800700. 30397036

[105] Singh M, Khong H, Dai Z, Huang X-F, Wargo JA, Cooper ZA, Vasilakos JP, Hwu P, Overwijk WW. Effective innate and adaptive antimelanoma immunity through localized TLR7/8 activation. J Immunol. 2014;193(9):4722–4731. doi:10.4049/jimmunol.1401160. 25252955 PMC4201984

[106] Khalil DN, Suek N, Campesato LF, Budhu S, Redmond D, Samstein RM, Krishna C, Panageas KS, Capanu M, Houghton S. In situ vaccination with defined factors overcomes T cell exhaustion in distant tumors. J Clin Invest. 2019;129(8):3435–3447. doi:10.1172/JCI128562. 31329159 PMC6668692

[107] Bellmann L, Cappellano G, Schachtl-Riess JF, Prokopi A, Seretis A, Ortner D, Tripp CH, Brinckerhoff CE, Mullins DW, Stoitzner P, et al. A TLR7 agonist strengthens T and NK cell function during BRAF-targeted therapy in a preclinical melanoma model. Int J Cancer. 2020;146(5):1409–1420. doi:10.1002/ijc.32777. 31702822 PMC7003881

[108] Mullins SR, Vasilakos JP, Deschler K, Grigsby I, Gillis P, John J, Elder MJ, Swales J, Timosenko E, Cooper Z, et al. Intratumoral immunotherapy with TLR7/8 agonist MEDI9197 modulates the tumor microenvironment leading to enhanced activity when combined with other immunotherapies. J Immunother Cancer. 2019;7(1):244. doi:10.1186/s40425-019-0724-8. 31511088 PMC6739946

[109] Etzerodt A, Tsalkitzi K, Maniecki M, Damsky W, Delfini M, Baudoin E, Moulin M, Bosenberg M, Graversen JH, Auphan-Anezin N, et al. Specific targeting of CD163. J Exp Med. 2019;216(10):2394–2411. doi:10.1084/jem.20182124. 31375534 PMC6781002

[110] Georgoudaki AM, Prokopec KE, Boura VF, Hellqvist E, Sohn S, Östling J, Dahan R, Harris R, Rantalainen M, Klevebring D, et al. Reprogramming tumor-associated macrophages by antibody targeting inhibits cancer progression and metastasis. Cell Rep. 2016;15(9):2000–2011. doi:10.1016/j.celrep.2016.04.084. 27210762

[111] Chen AX, Gartrell RD, Zhao J, Upadhyayula PS, Zhao W, Yuan J, Minns HE, Dovas A, Bruce JN, Lasorella A, et al. Single-cell characterization of macrophages in glioblastoma reveals MARCO as a mesenchymal pro-tumor marker. Genome Med. 2021;13(1):88. doi:10.1186/s13073-021-00906-x. 34011400 PMC8136167

[112] Eisinger S, Sarhan D, Boura VF, Ibarlucea-Benitez I, Tyystjärvi S, Oliynyk G, Arsenian-Henriksson M, Lane D, Wikström SL, Kiessling R, et al. Targeting a scavenger receptor on tumor-associated macrophages activates tumor cell killing by natural killer cells. Proc Natl Acad Sci U S A. 2020;117(50):32005–32016. doi:10.1073/pnas.2015343117. 33229588 PMC7750482

[113] Weiskopf K, Weissman IL. Macrophages are critical effectors of antibody therapies for cancer. MAbs. 2015;7(2):303–310. doi:10.1080/19420862.2015.1011450. 25667985 PMC4622600

[114] Zhang M, Wen B, Anton OM, Yao Z, Dubois S, Ju W, Sato N, DiLillo DJ, Bamford RN, Ravetch JV. IL-15 enhanced antibody-dependent cellular cytotoxicity mediated by NK cells and macrophages. Proc Natl Acad Sci U S A. 2018;115(46):E10915–E10924. doi:10.1073/pnas.1811615115. 30373815 PMC6243244

[115] Gül N, Babes L, Siegmund K, Korthouwer R, Bögels M, Braster R, Vidarsson G, ten Hagen TLM, Kubes P, van Egmond M, et al. Macrophages eliminate circulating tumor cells after monoclonal antibody therapy. J Clin Invest. 2014;124(2):812–823. doi:10.1172/JCI66776. 24430180 PMC3904600

[116] Wang H, Xu Q, Zhao C, Zhu Z, Zhu X, Zhou J, Zhang S, Yang T, Zhang B, Li J, et al. An immune evasion mechanism with IgG4 playing an essential role in cancer and implication for immunotherapy. J Immunother Cancer. 2020;8(2):e000661. doi:10.1136/jitc-2020-000661. 32819973 PMC7443307

[117] Karagiannis P, Villanova F, Josephs DH, Correa I, Van Hemelrijck M, Hobbs C, Saul L, Egbuniwe IU, Tosi I, Ilieva KM, et al. Elevated IgG4 in patient circulation is associated with the risk of disease progression in melanoma. Oncoimmunology. 2015;4(11):e1032492. doi:10.1080/2162402X.2015.1032492. 26451312 PMC4590000

[118] Karagiannis P, Gilbert AE, Josephs DH, Ali N, Dodev T, Saul L, Correa I, Roberts L, Beddowes E, Koers A, et al. IgG4 subclass antibodies impair antitumor immunity in melanoma. J Clin Invest. 2013;123(4):1457–1474. doi:10.1172/JCI65579. 23454746 PMC3613918

[119] Jordakieva G, Bianchini R, Reichhold D, Piehslinger J, Groschopf A, Jensen SA, Mearini E, Nocentini G, Crevenna R, Zlabinger GJ, et al. IgG4 induces tolerogenic M2-like macrophages and correlates with disease progression in colon cancer. Oncoimmunology. 2021;10(1):1880687. doi:10.1080/2162402X.2021.1880687. 33628623 PMC7889146

[120] Daveau M, Pavie-Fischer J, Rivat L, Rivat C, Ropartz C, Peter HH, Cesarini J-P, Kourilsky FM. IgG4 subclass in malignant melanoma. J Natl Cancer Inst. 1977;58(2):189–192. doi:10.1093/jnci/58.2.189. 833869

[121] Pellizzari G, Hoskin C, Crescioli S, Mele S, Gotovina J, Chiaruttini G, Bianchini R, Ilieva K, Bax HJ, Papa S. IgE re-programs alternatively-activated human macrophages towards pro-inflammatory anti-tumoural states. EBioMedicine. 2019;43:67–81. doi:10.1016/j.ebiom.2019.03.080. 30956175 PMC6562024

[122] Nakamura M, Souri EA, Osborn G, Laddach, R., Chauhan, J., Stavraka, C., Lombardi, S., Black, A., Khiabany, A., Khair, D O. et al, IgE Activates Monocytes from Cancer Patients to Acquire a Pro-Inflammatory Phenotype. Cancers (Basel). 2020 Nov;12(11). 3376. doi:10.3390/cancers12113376. PMC7698027 33203088

[123] Josephs DH, Bax HJ, Dodev T, Georgouli, M., Nakamura, M., Pellizzari, G., Saul, L., Karagiannis, P., Cheung, A., Herraiz, C. et al, Anti-Folate Receptor-α IgE but not IgG Recruits Macrophages to Attack Tumors via TNFα/MCP-1 Signaling. Cancer Research. 2017 03 01;77(5):1127–1141. doi:10.1158/0008-5472.CAN-16-1829. 28096174 PMC6173310

[124] Yu J, Song Y, Tian W. How to select IgG subclasses in developing anti-tumor therapeutic antibodies. J Hematol Oncol. 2020;13(1):45. doi:10.1186/s13045-020-00876-4. 32370812 PMC7201658

[125] White AL, Chan HT, Roghanian A, French RR, Mockridge CI, Tutt AL, Dixon SV, Ajona D, Verbeek JS, Al-Shamkhani A, et al. Interaction with FcγRIIB is critical for the agonistic activity of anti-CD40 monoclonal antibody. J Immunol. 2011;187(4):1754–1763. doi:10.4049/jimmunol.1101135. 21742972

[126] Mimoto F, Katada H, Kadono S, Igawa T, Kuramochi T, Muraoka M, Wada Y, Haraya K, Miyazaki T, Hattori K, et al. Engineered antibody Fc variant with selectively enhanced FcγRIIb binding over both FcγRIIa(R131) and FcγRIIa(H131). Protein Eng Des Sel. 2013;26(10):589–598. doi:10.1093/protein/gzt022. 23744091 PMC3785249

[127] Press MF, Cordon-Cardo C, Slamon DJ. Expression of the HER-2/neu proto-oncogene in normal human adult and fetal tissues. Oncogene. 1990;5(7):953–962. 1973830

[128] Huszno J, Leś D, Sarzyczny-Słota D, Nowara E. Cardiac side effects of trastuzumab in breast cancer patients - single center experiences. Contemp Oncol (Pozn). 2013;17(2):190–195. doi:10.5114/wo.2013.34624. 23788989 PMC3685367

[129] Budhu S, Giese R, Gupta A, Fitzgerald K, Zappasodi R, Schad S, Hirschhorn D, Campesato LF, De Henau O, Gigoux M. Targeting phosphatidylserine enhances the anti-tumor response to tumor-directed radiation therapy in a preclinical model of melanoma. Cell Rep. 2021;34(2):108620. doi:10.1016/j.celrep.2020.108620. 33440157 PMC8100747

[130] Yin Y, Huang X, Lynn KD, Thorpe PE. Phosphatidylserine-targeting antibody induces M1 macrophage polarization and promotes myeloid-derived suppressor cell differentiation. Cancer Immunol Res. 2013;1(4):256–268. doi:10.1158/2326-6066.CIR-13-0073. 24777853

[131] Mediavilla-Varela M, Page MM, Kreahling J, Freimark BD, Shan J, Kallinteris NL, Antonia SJ, Altiok S. Effect of bavituximab in combination with nivolumab on tumor immune response in a 3D ex vivo system of lung cancer patients. J Clin Oncol. 2017;35(15_suppl):e23091–e23091. doi:10.1200/JCO.2017.35.15_suppl.e23091.

[132] Chau I, Culm-Merdek K, Bendell JC, Catenacci DV, Lee J, Chaney MF, MacIntyre S, Gopal S, Santos VC, Youssoufian H, Mockbee C. 1386P - Phase II study of bavituximab (bavi), a first-in-class antibody targeting phosphatidylserine (PS), plus pembrolizumab (pembro) in advanced gastric or gastroesophageal junction (GEJ) cancer. Ann Oncol. 2021;32:S1040–S1075.

[133] Williams IP, Crescioli S, Sow HS, Bax HJ, Hobbs C, Ilieva KM, French E, Pellizzari G, Cox V, Josephs DH. In vivo safety profile of a CSPG4-directed IgE antibody in an immunocompetent rat model. MAbs. 2020;12(1):1685349. doi:10.1080/19420862.2019.1685349. 31769737 PMC6927758

