# Supplementary material for: Influencing tumor-associated macrophages in malignant melanoma with monoclonal antibodies
Source: Oncoimmunology. 2022 Oct 3;11(1):2127284. doi: 10.1080/2162402X.2022.2127284 (PMC9543025; doi:10.1080/2162402X.2022.2127284)
Supplement: Supplemental Material [file KONI_A_2127284_SM5447.docx]

| **Drug Name** | **Type** | **Target** | **Cancer Type** | **Phase** | **Outcome** | **Study Design** | **Route of drug administration** | **Reference** |
| --- | --- | --- | --- | --- | --- | --- | --- | --- |
| Emactuzumab | mAb (IgG1) | CSF1R | Solid tumours | 1 | Reduction of immunosuppressive TAMs  No clinically relevant anti-tumour activity | Emactuzumab +/- paclitaxel | Intravenous | NCT01494688 |
| Carlumab | mAb (IgG1) | CCL2 | Solid tumours | 1b | No long-term suppression of serum CCL2  No significant tumour responses were observed. | Carlumab + docetaxel or gemcitabine  or paclitaxel + carboplatin  or pegylated liposomal doxorubicin HCl | Intravenous | NCT00537368  NCT01204996 |
| Plozalizumab | mAb (IgG1) | CCR2 | Melanoma | 1b | Lack of discernible benefit | Plozalizumab + nivolumab | Oral | NCT02723006 |
| SGN-CD47M | ADC (IgG4) | CD47 | Solid tumours | 1 | No results | Monotherapy | Intravenous | NCT03957096 |
| TTI-621 | mAb (IgG1) | CD47 | Melanoma and other skin tumours | 1 | Some anti-tumour activity seen in adjacent or distal sites.  Closed to focus on intravenous studies of same drug | TTI-621 +/-  PD-1/PD-L1 inhibitor  or pegylated IFN-α2a or TVEC  or radiation | Intratumoural | NCT02890368 |

**Supplementary Table 1: Examples of monoclonal antibodies targeting macrophages that have failed to progress through clinical trials.** Data extracted from clinicaltrials.gov.

Abbreviations: monoclonal antibody, mAb; colony stimulating factor 1 receptor, CSF1R; antibody drug conjugate, ADC; programmed death 1/ligand 1, PD1-PD-L1.
